# Comparative efficacy of different Chinese patent medicines in preventing restenosis after percutaneous coronary intervention: a systematic review and Bayesian network meta-analysis of randomized clinical trials

**DOI:** 10.3389/fphar.2023.1265766

**Published:** 2024-01-05

**Authors:** Jiasai Fan, Tianli Li, Fenglan Pu, Nan Guo, Jing Wang, Yuqian Gao, Hongbing Zhao, Xian Wang, Haiyan Zhu

**Affiliations:** ^1^ Department of Cardiology, Dongzhimen Hospital, Beijing University of Chinese Medicine, Beijing, China; ^2^ National Integrated Traditional and Western Medicine Center for Cardiovascular Disease, China-Japan Friendship Hospital, Beijing, China; ^3^ Centre for Evidence-Based Chinese Medicine, Beijing University of Chinese Medicine, Beijing, China; ^4^ Dongzhimen Hospital, Beijing University of Chinese Medicine, Beijing, China; ^5^ Institute of Cardiovascular Diseases, Beijing University of Chinese Medicine, Beijing, China

**Keywords:** Chinese patent medicines, restenosis, percutaneous coronary intervention, efficacy, Bayesian network meta-analysis

## Abstract

**Background:** Chinese patent medicines (CMPs) have curative effectiveness in preventing coronary restenosis. However, the relative efficacy between different CPMs has not been sufficiently investigated.

**Methods:** Randomized clinical trials were searched from electronic databases including PubMed, Web of Science, Cochrane Library, Embase, CNKI, VIP, WanFang, SinoMed, Chinese Clinical Trial Registry, and ClinicalTrials.gov. Bayesian network meta-analysis was performed to analyze CPMs’ efficacy in preventing angiographic restenosis, recurrence angina, acute myocardial infarction, and target lesion revascularization after percutaneous coronary intervention.

**Results:** This network meta-analysis included 47 trials with 5,077 patients evaluating 11 interventions. Regarding angiographic restenosis, the efficacy of CPMs (except Xuezhikang capsule) combined with standard treatment (Std) was superior to Std alone, and Guanxin Shutong capsule plus Std reduced the risk of angiographic restenosis by 76% (relative risk 0.24, 95% confidence interval 0.11–0.45, and very low to moderate certainty of evidence), most likely the best intervention. Fufang Danshen dripping pill combined with Std showed superiority over other interventions for relieving recurrence angina, which can reduce the risk by 83% (RR 0.17, 95% CI 0.04–0.51, very low to moderate certainty of evidence) compared to Std alone. In acute myocardial infarction after percutaneous coronary intervention, compared with Std alone, Danhong injection plus Std displayed a significant effect (RR 0.11, 95% CI 0.00–0.69, very low to moderate certainty of evidence) and was the best treatment probably. Chuanxiongqin tablet plus Std was the most effective treatment for reducing target lesion revascularization by 90% (RR 0.10, 95% CI 0.00–0.60, very low to moderate certainty of evidence) compared with Std alone.

**Conclusion:** The results indicated that CPMs combined with Std reduced the risk of coronary restenosis after percutaneous coronary intervention. However, the results should be interpreted cautiously due to significant data limitations.

## 1 Introduction

Percutaneous coronary intervention (PCI) improves myocardial perfusion by dredging a narrow or even occluded coronary artery lumen through the cardiac catheterization technique (Grines et al., 2016). However, due to the complex pathological mechanism of thrombosis, intimal hyperplasia, and inflammatory response, patients undergoing PCI have a risk of coronary restenosis (Jukema et al., 2012; Melnik et al., 2022). Importantly, PCI for restenosis is associated with a higher risk of major adverse cardiac events than PCI for *de novo* lesions (Giustino et al., 2022). Although standard treatment (Std) recommended by the guideline (Lawton et al., 2022), including aspirin, clopidogrel, and statin, has been shown to reduce restenosis risk, in-stent restenosis still occurs at a rate of 5% in patients with PCI (Moussa et al., 2020). Given that millions of people undergo PCI treatment annually worldwide (Tsao et al., 2022; Chinese Cardiovascular Health and Disease report writing group, 2023), restenosis can be considered a significant public health problem.

As an adjunct drug, traditional Chinese medicine (TCM) combined with Std has a specific curative effectiveness in preventing restenosis (Wu et al., 2019). Among TCM, many clinical trials on Chinese patent medicines (CPMs) in preventing restenosis have been conducted due to the advantages of standardized dosage and composition, stable and controllable quality, and convenient taking (Xu et al., 2000; Li et al., 2004; Mao et al., 2015; Zhou et al., 2021). However, the relative efficacy between different CPMs has not been sufficiently investigated.

Network meta-analysis (NMA), a novel meta-analysis strategy, can integrate direct and indirect evidence. It allows comparisons across multiple treatments simultaneously even if they were not directly compared previously. Furthermore, a Bayesian approach to NMA provides the probability estimates that enable clinicians to select the optimal treatment option (Sutton and Abrams, 2001). The objective of this study was to evaluate the comparative effectiveness of CPMs in preventing coronary restenosis after PCI using a Bayesian NMA approach.

## 2 Materials and methods

We followed the Preferred Reporting Items for Systematic Reviews and Meta-Analyses (PRISMA) and the extension statement for network meta-analysis (PRISMA-NMA) to report the current results (Hutton et al., 2015; Page et al., 2021).

### 2.1 Standard evaluation of CMPs

To make this study reproducible, we reported CMPs according to the requirements of the ConPhyMP consensus (Heinrich et al., 2020). Accurate scientific nomenclature for botanical drugs referred to Rivera’s suggestion (Rivera et al., 2014) and was validated taxonomically in the databases of “Medicinal Plant Names Services” (https://mpns.science.kew.org/mpns-portal/). In addition, we referred to the Chinese Pharmacopoeia 2020 regarding the names of non-botanical drugs. The relevant information about CMPs referred to the original study, the Chinese Pharmacopoeia 2020, and the National Medical Products Administration. The details are shown in Supplementary Tables S1, S2.

### 2.2 Inclusion criteria

We determined the literature to be included according to the PICOS principle: 1) patients: who accepted the treatment of PCI. 2) Intervention: the treatment group received Std for coronary heart disease (including aspirin, clopidogrel, statin, angiotensin-converting enzyme inhibitor or angiotensin receptor blocker, and β-blocker) and additional CPMs. 3) Comparison: the control group received a Std treatment for coronary heart disease, with or without a placebo. 4) Outcome: the angiographic restenosis rate, defined as target vessel lumen stenosis ≥50% according to the angiographic results. 5) Study design: randomized controlled trial (RCT).

### 2.3 Exclusion criteria

We excluded duplicate literature, research lacking important data, articles in which the full text was not found, published papers using the same data, and trials followed for less than 4 weeks.

### 2.4 Sources and search strategy of the literature

We searched PubMed, Web of Science, Cochrane Library, Embase, CNKI, VIP, WanFang, and SinoMed from these electronic databases’ inception to 13 April 2023. In addition, two clinical trial registration platforms, namely, Chinese Clinical Trial Registry (https://www.chictr.org.cn/) and ClinicalTrials.gov (https://clinicaltrials.gov/), were also searched. A detailed search strategy for English databases can be seen in Supplementary Texts S1–S4.

### 2.5 Literature screening and data extraction

All studies were screened and data were extracted independently by two researchers. First, we used EndNote X9 software to remove duplicates. Then, two researchers read the title and abstract to finish the preliminary screening and finally read the full text to decide whether a study was included appropriately. In case of disagreement, the third researcher helped resolve the problem.

Data extraction still adopted the double entry and cross-check method. We tried to contact the authors to acquire missing information when we encountered incomplete data. Data extraction included title, author, disease, sample size, age, sex, intervention measures, course of treatment, outcome indicators, and follow-up time.

### 2.6 Bias risk and GRADE certainty assessment

The Cochrane Risk of Bias 2 tool (Cochrane Collaboration, London, United Kingdom) (Sterne et al., 2019) was used to assess the risk of bias of the included studies by two researchers. The tool assesses bias across six distinct domains: randomization process, deviations from intended interventions, missingness in outcome data, measurement of the outcome, selection of reported results, and overall bias. The judgment of each domain includes low risk, some concerns, and high risk. We used the GRADE approach for the entire network to provide the framework for rating the certainty of the evidence of each paired comparison as high, moderate, low, or very low (Puhan et al., 2014; Brignardello-Petersen et al., 2020).

### 2.7 Outcomes

The primary outcome was the angiographic restenosis rate defined as target vessel lumen stenosis ≥50% according to the angiographic results. Furthermore, we focused on the secondary outcomes, including recurrence angina, acute myocardial infarction (AMI) after PCI, and target lesion revascularization (TLR), defined as clinical restenosis.

### 2.8 Statistical analysis

NMA estimating the treatment effectiveness was conducted using Bayesian Markov chain Monte Carlo algorithms. The outcomes in this study were all categorical variables, and the statistical analysis results were expressed as relative risk (RR) with 95% confidence intervals (CIs). To account for clinical and methodological heterogeneity among studies when comparing treatment effectiveness, random-effects models were selected. The heterogeneity of the entire network was estimated using a global I^2^ statistic.

Models were calculated by generating 50,000 sample iterations with an initial burn-in period of 20,000 iterations (thin = 1). Four chains with different initial values were run simultaneously to assess convergence using the trace and density and Brooks–Gelman–Rubin diagnostic plots. The potential scale reduction factor value of the Brooks–Gelman–Rubin diagnostics close to 1 indicates approximate convergence. The surface under the cumulative ranking (SUCRA) curve value was calculated based on the evaluation of rank probabilities. No consistency evaluation was performed because all data were from indirect treatment comparisons and no head-to-head RCTs.

We performed subgroup network meta-analysis for angiographic restenosis according to the number of patients included in the original study (≥100 or <100). Sensitivity analysis was carried out by excluding studies with a high-risk bias. All statistical analyses of the NMA were conducted using the “gemtc” package in R v4.0.2 software (R Project; www.r-project.org). In addition, plots of network diagrams visualizing network geometry and node connectivity and funnel plots examining publication bias were created using Stata/SE 15.1.

## 3 Results

### 3.1 Study selection

**FIGURE 1:**
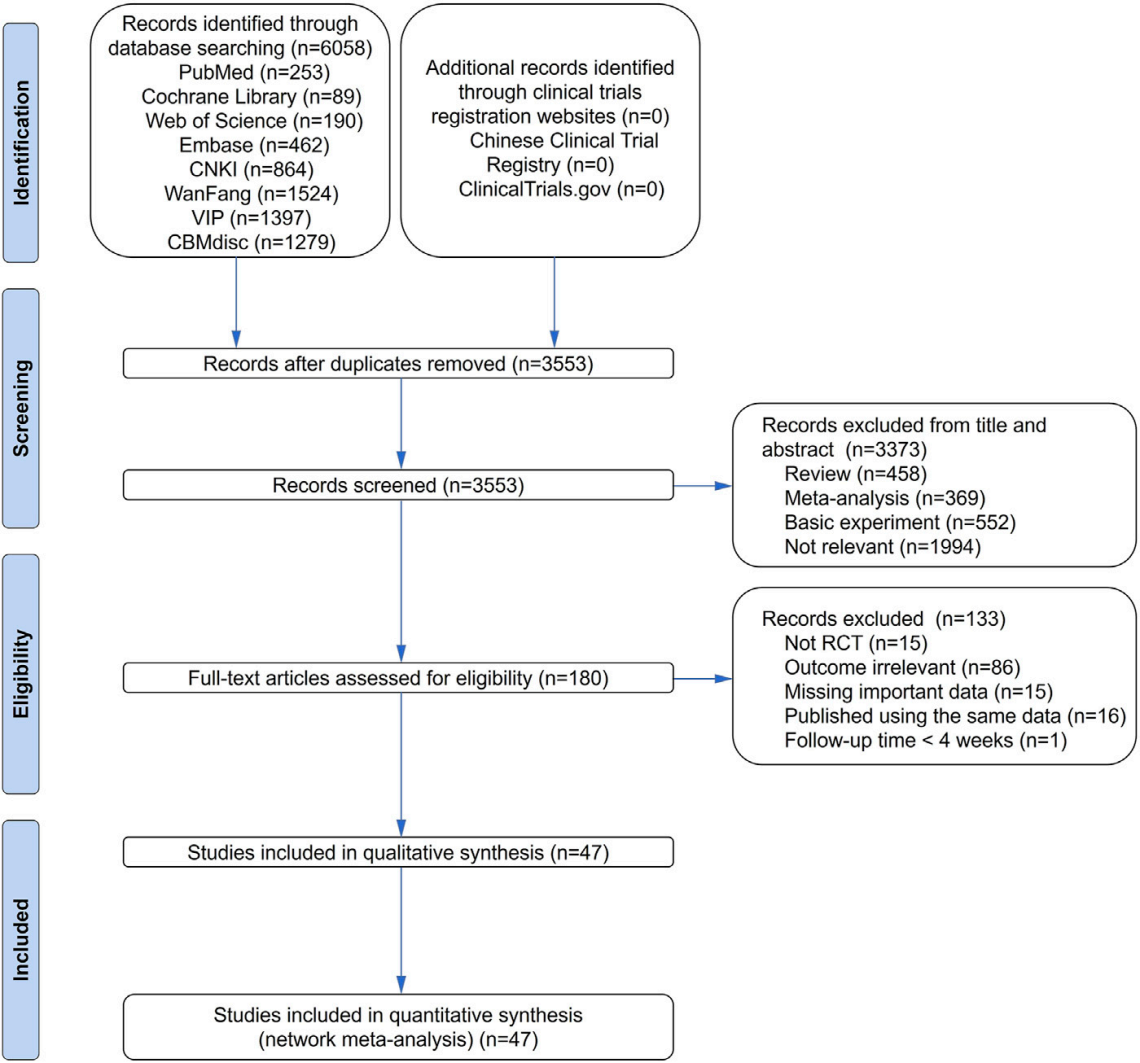
Summary of trial identification and selection. RCT, randomized controlled trial; TCM, traditional Chinese medicine.

A total of 6,058 articles were found through the electronic database search, including 253 from PubMed, 89 from the Cochrane Library, 190 from the Web of Science, 462 from Embase, 864 from CNKI, 1,524 from WanFang, 1,397 from VIP, and 1,279 from CBMdisc. Relevant trials were not found from Chinese Clinical Trial Registry and ClinicalTrials.gov. After combining these articles, 2,505 duplicates were removed. After removing duplicates, 3,553 articles were screened for the title and abstract, of which 3,373 were excluded. Afterward, 180 relevant articles were screened for eligibility by reading the full text. Finally, 47 articles (Supplementary Text S5) that met the inclusion criteria were included in our Bayesian NMA. The details of the literature screening process are shown in Figure 1.

### 3.2 Study characteristics

The Bayesian NMA was conducted including 47 studies that enrolled a total of 5,077 patients. All trials were conducted in China. One of them was written in English, and 46 were written in Chinese. NMA evaluated 11 different treatments in preventing restenosis: Std, Tongxinluo capsule plus Std (represented as TXLC), Danhong injection plus Std (represented as DHI), Qishen Yiqi dripping pill plus Std (represented as QYDP), Guanxin Shutong capsule plus Std (represented as GXSTC), Shexiang Baoxin pill plus Std (represented as SBP), Xiongshao capsule plus Std (represented as XSC), Chuanxiongqin tablet plus Std (represented as CXQ), Guanxin Tongluo capsule plus Std (represented as GXTLC), Fufang Danshen dripping pill plus Std (represented as FDDP), and Xuezhikang capsule plus Std (represented as XZK). The mean (or median) age of the patients ranged from 50 to 72 years, and the follow-up time varied from 3 to 24 months (Table 1).

**TABLE 1:** Summary of included clinical trials and patient characteristics.

Trial	Disease	No. of patients		Age (years)		Sex (male, %)		Treatment		Period of treatment	Follow-up
		Intervention group	Control group	Intervention group	Control group	Intervention group	Control group	Intervention group	Control group		
Zhou and Guo (2007)	CHD	70	66	56.8 (30.0–76.0)		63.2		Std + TXLC (three capsules, tid, 6 m)	Std	6 m	6 m
Liu and Jiang (2017)	ACS	52	52	57.3 ± 19.6	57.3 ± 19.6	69.2		Std + TXLC (three capsules, tid, 6 m)	Std	6 m	6 m
Lu et al. (2014)	CHD	90	90	60.2 ± 6.9	61.8 ± 7.2	53.3	51.1	Std + TXLC (three capsules, tid, 12 m)	Std + Pbo	12 m	12 m
Liu (2019)	CHD	55	55	60.5 ± 8.6	61.0 ± 8.4	60.0	63.6	Std + TXLC (three capsules, tid, 1 m)	Std	1 m	6 m
Tian (2017)	CHD	58	58	61.2 ± 6.3	61.0 ± 6.3	69.0	67.2	Std + TXLC (three capsules, tid, 6 m)	Std	6 m	6 m
Li (2020)	ACS	43	43	61.7 ± 6.9	62.5 ± 6.5	69.8	67.4	Std + TXLC (three capsules, tid, 6 m)	Std	6 m	6 m
Yao et al. (2006)	CHD	38	38	56.6 ± 10.9	50.1 ± 12.4	57.9		Std + TXLC (four capsules, tid, 6 m)	Std	6 m	6 m
Deng (2013)	CHD	31	30	54.8 ± 3.1	55.2 ± 3.0	67.7	66.7	Std + TXLC (four capsules, tid, 6 m)	Std	6 m	6 m
Dai and Yang (2011)	CHD	31	30	61.3 ± 7.23	63.5 ± 6.1	61.3	56.7	Std + TXLC (6 m)	Std	6 m	6 m
Wang and Shi (2009)	CHD	40	40	54.2 ± 10.2	52.5 ± 11.0	70.0	65.0	Std + TXLC (three capsules, tid, 6 m)	Std	6 m	6 m
Liang et al. (2010)	AMI	42	38	55.0 (40.0–70.0)	55.0 (40.0–70.0)	65.0		Std + TXLC (two capsules, tid, 6 m)	Std	6 m	6 m
Di (2012)	CHD	25	25	64.6 ± 8.7	63.0 ± 7.8	72.0	64.0	Std + TXLC (three capsules, tid, 6 m)	Std	6–12 m	6–12 m
Sun and Lang (2018)	CHD	67	67	62.7 ± 3.1	60.8 ± 7.8	65.7	68.7	Std + TXLC (three capsules, tid, 6 m)	Std	6 m	6 m
Wang and Lou (2010)	CHD	68	64	65.2 ± 9.9	65.1 ± 10.4	63.2	70.3	Std + TXLC (three capsules, tid, 12 m)	Std + Pbo	12 m	12 m
Xiao et al. (2007)	CHD	62	70	53.0 ± 12.0	55.0 ± 10.0	67.7	70.0	Std + TXLC (four capsules, tid, 6 m)	Std	6 m	6 m
Han (2018)	CHD	34	34	59.3 ± 6.5	59.4 ± 6.6	61.8	64.7	Std + TXLC (four capsules, tid, 6 m)	Std	6 m	6 m
Qin (2018)	CHD	50	50	59.0 ± 3.5	58.3 ± 2.1	66.0	64.0	Std + DHI (40 mg/d, 10 days)	Std	10 days	6 m
Cui et al. (2021)	AMI	45	45	57.5 ± 3.4	56.4 ± 3.2	46.7	55.6	Std + DHI (30 mL, qd, 2 m)	Std	2 m	6 m
Luan (2011)	CHD	32	46	62.1 ± 11.9	61.9 ± 13.2	65.6	69.6	Std + DHI (20 mL, qd, 2 w)	Std	2 w	6 m
Chen et al. (2015)	ACS	60	60	61.4 ± 8.6	61.5 ± 9.4	60.0	65.0	Std + DHI (40 mL, qd, 2 w)	Std	2 w	6 m
Wang et al. (2011)	CHD	84	84	57.8 ± 10.2	58.6 ± 9.7	77.4	79.8	Std + DHI (40 mL, qd, 10 days)	Std	6 m	6 m
Tian et al. (2012)	CHD	39	36	—	—	—	—	Std + QYDP (10 pills, tid, 6 m)	Std	6 m	6 m
Li et al. (2014)	AMI	43	40	53.1 ± 9.5	54.2 ± 10.7	62.8	55.0	Std + QYDP (0.5 g, tid, 9 m)	Std	9 m	9 m
Wang (2013)	ACS	50	50	60.8 ± 8.9	60.6 ± 9.5	56.0	46.0	Std + QYDP (0.5 g, tid, 6 m)	Std	6 m	6 m
Li (2014)	CHD	55	55	61.2 ± 5.8	60.6 ± 5.2	69.1	65.6	Std + QYDP (0.5 g, tid, 6 m)	Std	6 m	6 m
Tu et al. (2021)	ACS	60	60	63.6 ± 8.4	66.0 ± 8.4	71.7	76.7	Std + QYDP (0.5 g, tid, 6 m)	Std	6 m	12 m
Zhuang et al. (2019)	AMI	54	54	52.1 ± 5.8	54.1 ± 4.2	44.4	57.4	Std + GXSTC (three capsules, tid, 12 m)	Std	12 m	12 m
Liu and Hou (2017)	AMI	14	16	58.2 ± 11.8	58.2 ± 11.8	78.6	87.5	Std + GXSTC (three capsules, tid, 12 m)	Std	12 m	12 m
Huang (2018)	AMI	46	46	59.9 ± 5.1	59.5 ± 5.0	58.7	52.2	Std + GXSTC (three capsules, tid, 6 m)	Std	6 m	6 m
Wang et al. (2014)	CHD	51	51	62.0 ± 8.6	61.3 ± 8.1	70.6	66.7	Std + GXSTC (three capsules, tid, 6 m)	Std	6 m	6 m
Chen et al. (2008)	CHD	95	81	57.0 ± 8.7	57.0 ± 8.7	63.6		Std + SBP (two pills, tid, 6 m)	Std	6 m	6 m
Yang et al. (2014)	CHD	103	90	72.2 ± 10.3	72.2 ± 10.3	68.8		Std + SBP (two pills, tid, 12 m)	Std	13 m	12 m
Huang et al. (2019)	AMI	30	30	56.8 ± 12.6	55.1 ± 12.3	56.7	60.0	Std + SBP (two pills, tid, 12 m)	Std	12 m	12 m
Si and Jia (2013)	CHD	60	60	50.1 ± 11.3	50.1 ± 11.3	—	—	Std + SBP (two pills, tid, 6 m)	Std	6 m	18 m
Kang (2017)	CHD	49	44	68.3 ± 2.8	67.8 ± 2.4	55.1	52.3	Std + XSC (500 mg, tid, 1 m)	Std	1 m	6–12 m
Xu et al. (2000)	CHD	28	37	57.8 ± 10.4	59.2 ± 10.6	82.1	83.8	Std + XSC (500 mg, tid, 6 m)	Std	6 m	6 m
Liu (2019)	CHD	41	41	62.8 (48.0–76.0)	62.8 (48.0–76.0)	54.9		Std + XSC (6 m)	Std	6 m	6–24 m
Chen et al. (2006)^a^	CHD	157	157	58.5 ± 10.3	58.7 ± 9.9	79.0	78.3	Std + XSC (500 mg, tid, 6 m)	Std + Pbo	6 m	6 m
Xiao et al. (2010)	CHD	42	38	55.6 (41.0–75.0)	55.6 (41.0–75.0)	55.0		Std + CXQ (50 mg, tid, 6 m)	Std	6 m	6 m
Pan (2016)	CHD	45	45	52.3 ± 0.5	52.65 ± 0.43	48.9	51.1	Std + CXQ (500 mg/d, 6 m)	Std	6 m	6 m
Liu et al. (2004)	CHD	63	102	55.5 ± 10.1	55.5 ± 10.1	81.0	81.4	Std + CXQ (50 mg, tid, 6 m)	Std	6 m	6 m
Li et al. (2016)	CHD	34	34	60.2 ± 4.3	61.3 ± 5.2	58.8	55.9	Std + GXTLC (1.6 g, tid, 6 m)	Std	6 m	6 m
Zhu et al. (2016)	CHD	42	40	70.4 ± 2.2	67.8 ± 2.4	57.1	57.5	Std + GXTLC (three pills, tid, 3 m)	Std	3 m	3 m
Dong (2019)	CHD	45	45	64.0 ± 3.0	65.0 ± 3.0	57.8	55.6	Std + GXTLC (1.6 g, tid, 3 m)	Std	3 m	3 m
Li et al. (2010)	CHD	40	40	52.6 (32.0–75.0)	52.6 (32.0–75.0)	55.5		Std + FDDP (10 pills, tid, 6 m)	Std	6 m	6 m
Tang and Zhang (2015)	CHD	44	44	53.6 ± 2.4	53.0 ± 2.2	65.9	70.5	Std + FDDP (10 pills, tid, 6 m)	Std	6 m	6 m
Zhao et al. (2015)	AMI	136	113	61.3 ± 12.1	62.5 ± 12.5	84.1		Std + XZK (0.6 g, bid, 6 m)	Std	9 m	9 m

ACS, acute coronary syndrome; AMI, acute myocardial infarction; CHD, coronary heart disease; CXQ, Chuanxiongqin tablet; DHI, Danhong injection; FDDP, Fufang Danshen dripping pill; GXSTC, Guanxin Shutong capsule; GXTLC, Guanxin Tongluo capsule; QYDP, Qishen Yiqi dripping pill; SBP, Shexiang Baoxin pill; Std, standard treatment; TXLC, Tongxinluo capsule; XSC, Xiongshao capsule; XZK, Xuezhikang capsule; d, day; m, month; w, week.

^a^ Written in English.

### 3.3 Risk of bias assessment

**FIGURE 2:**
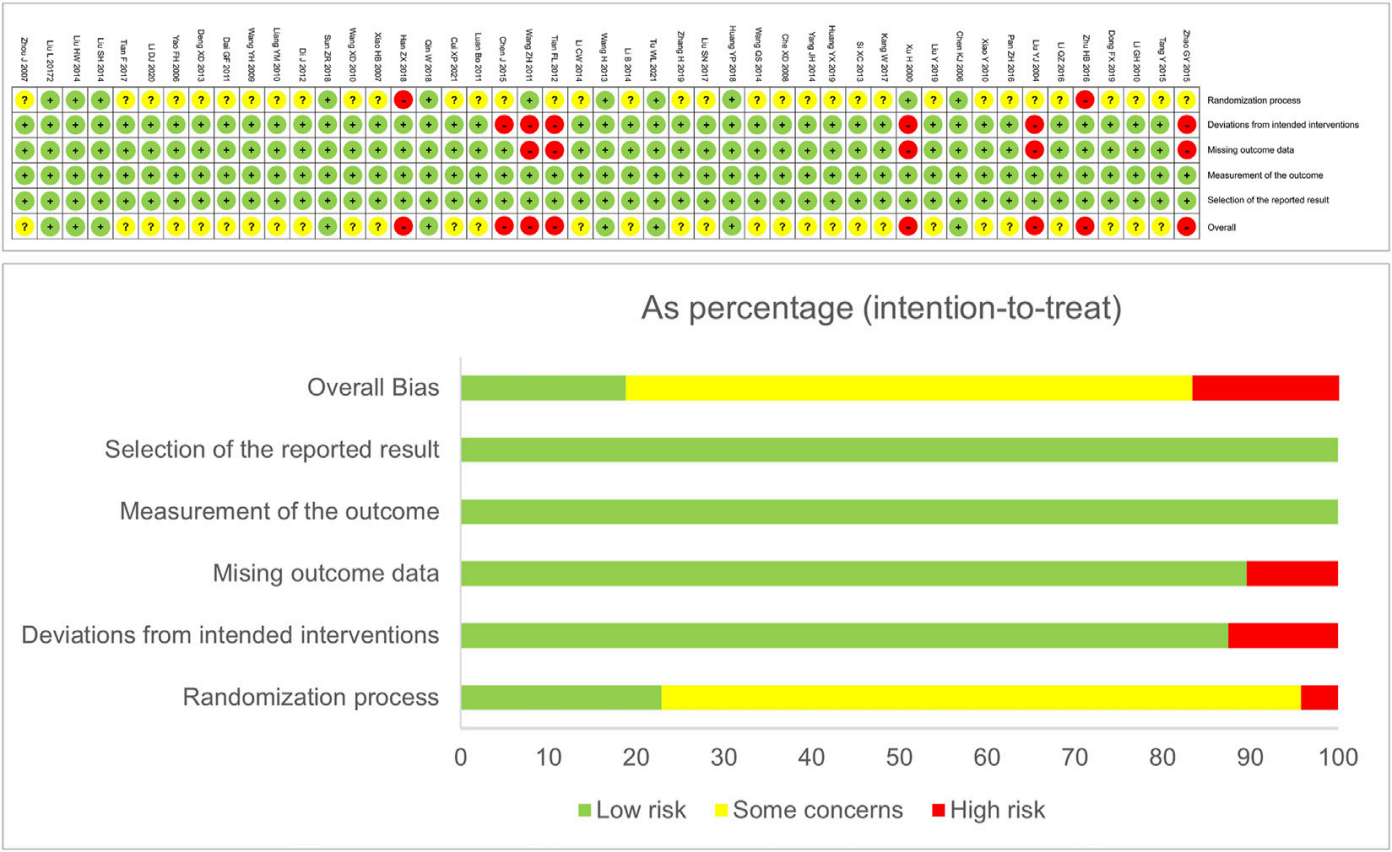
Risk of bias summary and risk of bias graph.

In terms of the randomization process, 11 studies had clear random sequence generation, 34 studies mentioned “random” only and did not describe the random method, and two studies enrolled patients according to their order of the first visit. Only three studies were double-blind trials. Six studies failed to follow up with all patients and did not mention appropriate analysis to estimate the effect of assignment to intervention. Five studies missed complete outcome data. All studies had an appropriate method of measuring the outcomes and did not have the risk of bias in selection of the reported result. The details of bias risk assessment are shown in Figure 2 and Supplementary Excel.

### 3.4 Assessment of heterogeneity and model fitting

For the outcomes of angiographic restenosis and TLR, there was heterogeneity in comparison pairs between XSC and Std (I^2^ = 69.8%) and DHI and Std (I^2^ = 50.6%). No heterogeneity was observed for the outcomes of recurrence angina and AMI after PCI.

The trace and density and Brooks–Gelman–Rubin diagnostic plots are shown in Supplementary Figures S1, S2. In the trace and density plot, the Markov chain Monte Carlo chain fluctuated steadily with good overlap, and the curve was smooth; in addition, the potential scale reduction factor value was close to 1. The above results showed that the model had a strong degree of convergence.

### 3.5 Primary outcome: angiographic restenosis

#### 3.5.1 Traditional pairwise meta-analysis

A traditional pairwise meta-analysis of the included study data was performed. There were 11 different interventions and 10 pairwise comparisons generated. All CPMs combined with Std except XZK were superior to Std alone (Supplementary Figure S3).

#### 3.5.2 Evidence network

**FIGURE 3:**
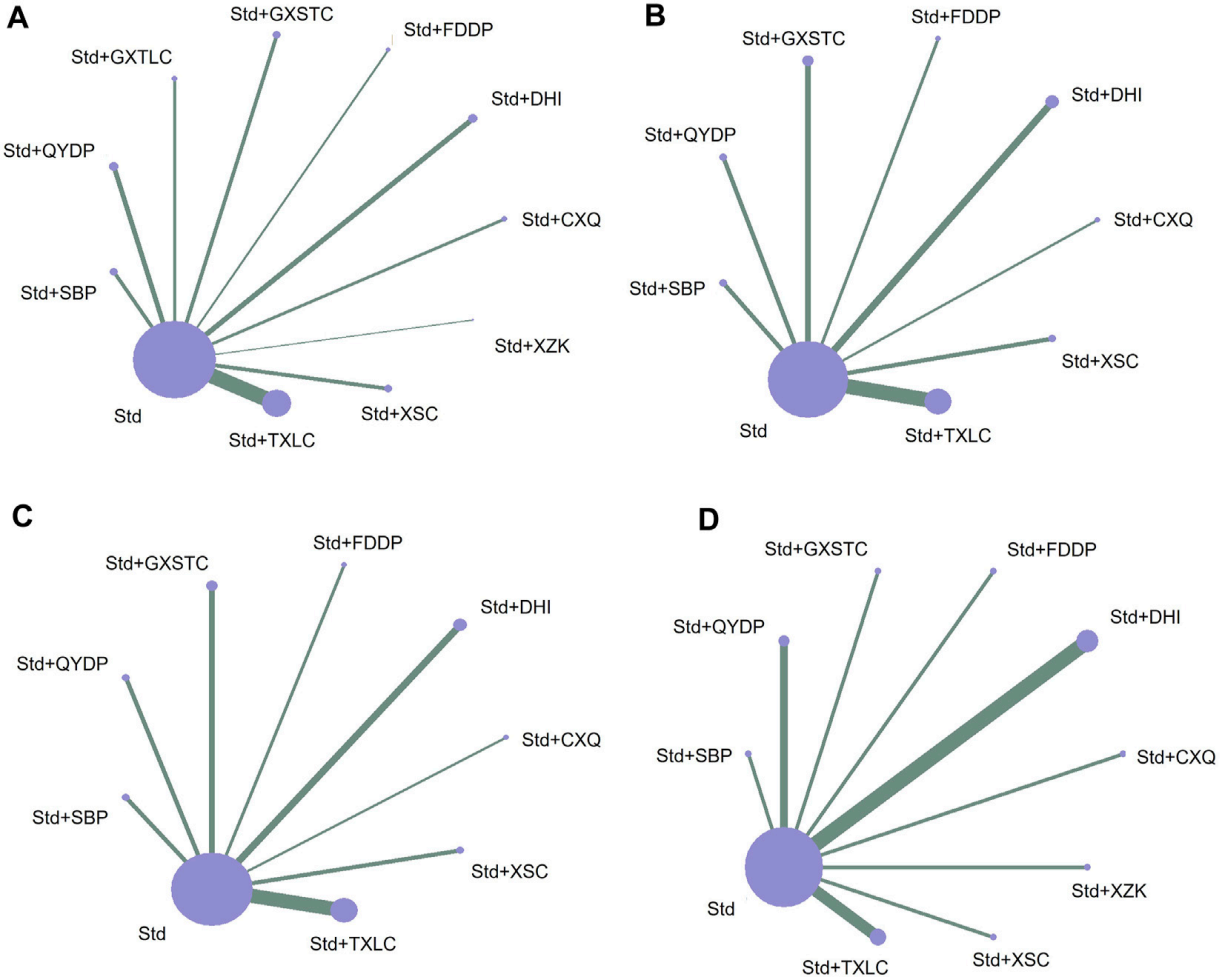
Network of treatment comparisons for Bayesian network meta-analysis. **(A)** Outcome of angiographic restenosis; **(B)** outcome of recurrence angina; **(C)** outcome of acute myocardial infarction; and **(D)** outcome of target lesion revascularization. CXQ, Chuanxiongqin tablet; DHI, Danhong injection; FDDP, Fufang Danshen dripping pill; GXSTC, Guanxin Shutong capsule; GXTLC, Guanxin Tongluo capsule; QYDP, Qishen Yiqi dripping pill; SBP, Shexiang Baoxin pill; Std, standard treatment; TXLC, Tongxinluo capsule; XSC, Xiongshao capsule; XZK, Xuezhikang capsule.

In total, 47 studies including 11 medication strategies involving 5,077 patients reported angiographic restenosis. The studies provided 10 direct comparisons (Figure 3A). Line thickness matches the number of included trials, and the circle size is related to the number of included patients. The number of studies that compared TXLC with Std and the number of patients who received the treatment were 16 and 1,606, respectively, and these were the largest numbers compared with other direct comparisons.

#### 3.5.3 NMA and SUCRA

**FIGURE 4:**
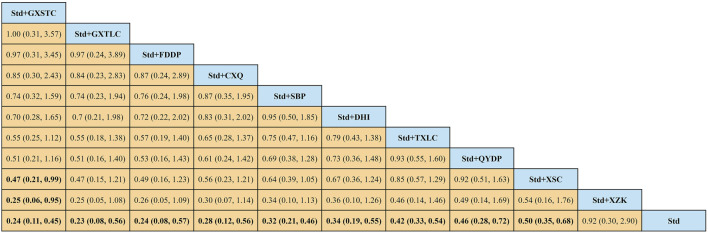
Summary of results of angiographic restenosis from network meta-analysis. CXQ, Chuanxiongqin tablet; DHI, Danhong injection; FDDP, Fufang Danshen dripping pill; GXSTC, Guanxin Shutong capsule; GXTLC, Guanxin Tongluo capsule; QYDP, Qishen Yiqi dripping pill; SBP, Shexiang Baoxin pill; Std, standard treatment; TXLC, Tongxinluo capsule; XSC, Xiongshao capsule; XZK, Xuezhikang capsule.

**FIGURE 5:**
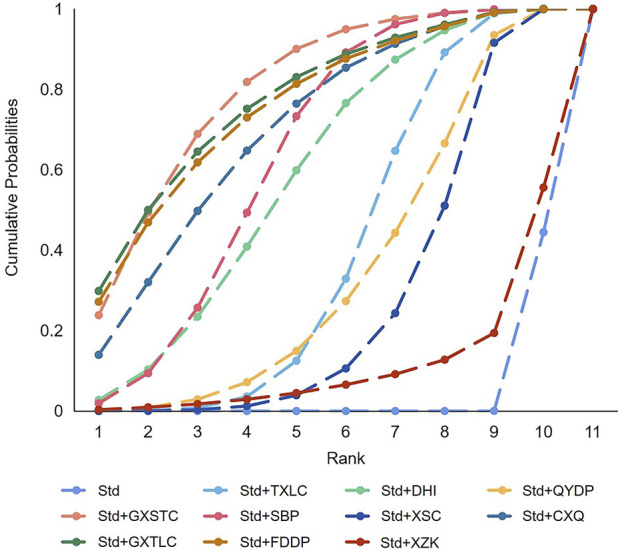
SUCRA curve with regard to reducing angiographic restenosis. CXQ, Chuanxiongqin tablet; DHI, Danhong injection; FDDP, Fufang Danshen dripping pill; GXSTC, Guanxin Shutong capsule; GXTLC, Guanxin Tongluo capsule; QYDP, Qishen Yiqi dripping pill; SBP, Shexiang Baoxin pill; Std, standard treatment; TXLC, Tongxinluo capsule; XSC, Xiongshao capsule; XZK, Xuezhikang capsule; SUCRA, surface under the cumulative ranking.

A total of 55 pairwise comparisons of angiographic restenosis were generated by NMA (Figure 4). In addition to nine pairwise direct comparisons, two pairwise indirect comparisons were also statistically significant. Compared with XSC and XZK, GXST reduced the risk of angiographic restenosis by 53% (RR 0.47, 95% CI 0.21–0.99) and 75% (RR 0.25, 95% CI 0.06–0.95), respectively. As shown in Figure 5 and Supplementary Table S3 for SUCRA of angiographic restenosis, GXST was most likely the best intervention. Certainty of evidence for all comparisons was very low to moderate. The details of evidence evaluation are available in Supplementary Table S4.

### 3.6 Secondary outcome: recurrence angina

#### 3.6.1 Traditional pairwise meta-analysis

A traditional pairwise meta-analysis of the included study data was performed. There were nine different interventions and eight pairwise comparisons generated. All CPMs combined with Std were superior to Std alone (Supplementary Figure S4).

#### 3.6.2 Evidence network

In total, 33 studies including nine medication strategies involving 3,749 patients reported recurrence angina. The studies provided eight direct comparisons (Figure 3B). The number of studies that compared TXLC with Std and the number of patients who received the treatment were 11 and 1,214, respectively.

#### 3.6.3 NMA and SUCRA

**FIGURE 6:**
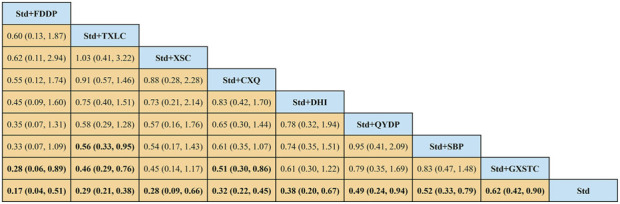
Summary of results of recurrence angina from network meta-analysis. CXQ, Chuanxiongqin tablet; DHI, Danhong injection; FDDP, Fufang Danshen dripping pill; GXSTC, Guanxin Shutong capsule; QYDP, Qishen Yiqi dripping pill; SBP, Shexiang Baoxin pill; Std, standard treatment; TXLC, Tongxinluo capsule; XSC, Xiongshao capsule.

**FIGURE 7:**
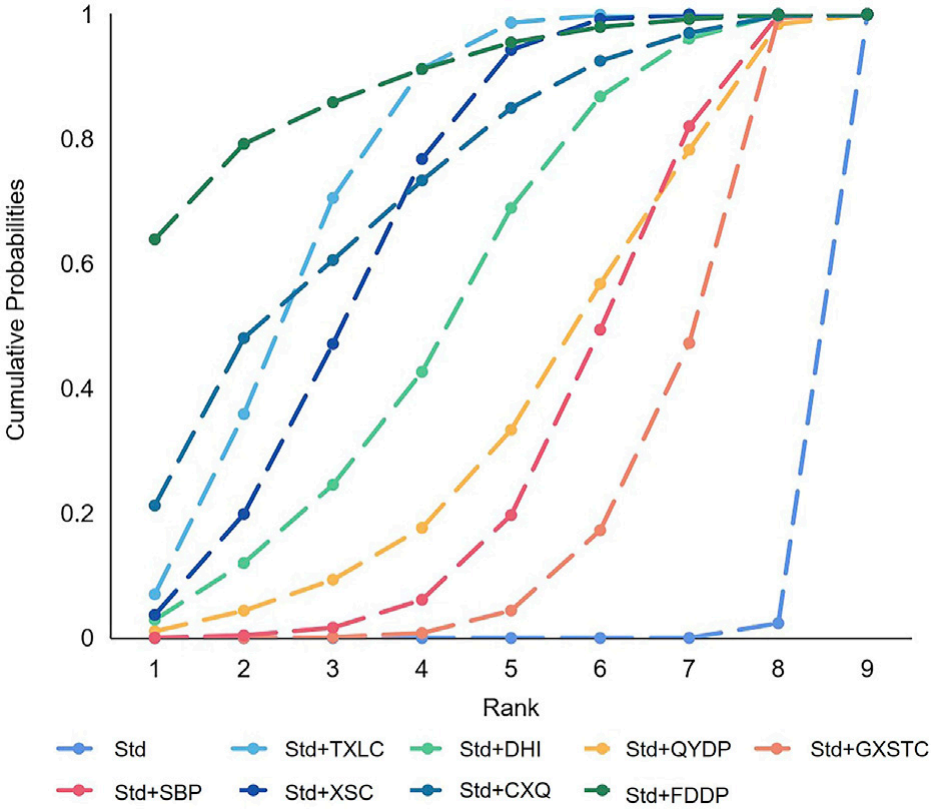
SUCRA curve with regard to reducing recurrence angina. CXQ, Chuanxiongqin tablet; DHI, Danhong injection; FDDP, Fufang Danshen dripping pill; GXSTC, Guanxin Shutong capsule; QYDP, Qishen Yiqi dripping pill; SBP, Shexiang Baoxin pill; Std, standard treatment; TXLC, Tongxinluo capsule; XSC, Xiongshao capsule; SUCRA, surface under the cumulative ranking.

A total of 36 pairwise comparisons of recurrence angina were generated by NMA (Figure 6). In addition to eight pairwise direct comparisons, four pairwise indirect comparisons were also statistically significant. Compared with GXSTC, FDDP, and TXLC, CXQ reduced the risk of recurrence angina by 72% (RR 0.28, 95% CI 0.06–0.89), 54% (RR 0.46, 95% CI 0.29–0.76), and 49% (RR 0.51, 95% CI 0.30–0.86), respectively. As shown in Figure 7 and Supplementary Table S3 for the SUCRA of recurrence angina, FDDP was most likely the best intervention. Certainty of evidence for all comparisons was very low to moderate. The details of evidence evaluation are available in Supplementary Table S5.

### 3.7 Secondary outcome: AMI

#### 3.7.1 Traditional pairwise meta-analysis

A traditional pairwise meta-analysis of the included study data was performed. There were nine different interventions and eight pairwise comparisons generated. Only DHI or TXLC was superior to Std (Supplementary Figure S5).

#### 3.7.2 Evidence network

In total, 17 studies including nine medication strategies involving 2,143 patients reported AMI. The studies provided eight direct comparisons (Figure 3C). The number of studies that compared TXLC with Std and the number patients who received the treatment were 7 and 844, respectively, and these are the largest numbers compared with other direct comparisons.

#### 3.7.3 NMA and SUCRA

**FIGURE 8:**
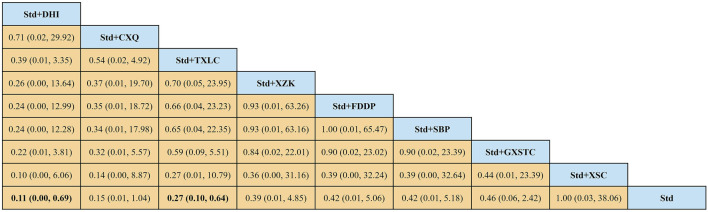
Summary of results of acute myocardial infarction from network meta-analysis. CXQ, Chuanxiongqin tablet; DHI, Danhong injection; FDDP, Fufang Danshen dripping pill; GXSTC, Guanxin Shutong capsule; SBP, Shexiang Baoxin pill; Std, standard treatment; TXLC, Tongxinluo capsule; XSC, Xiongshao capsule; XZK, Xuezhikang capsule.

**FIGURE 9:**
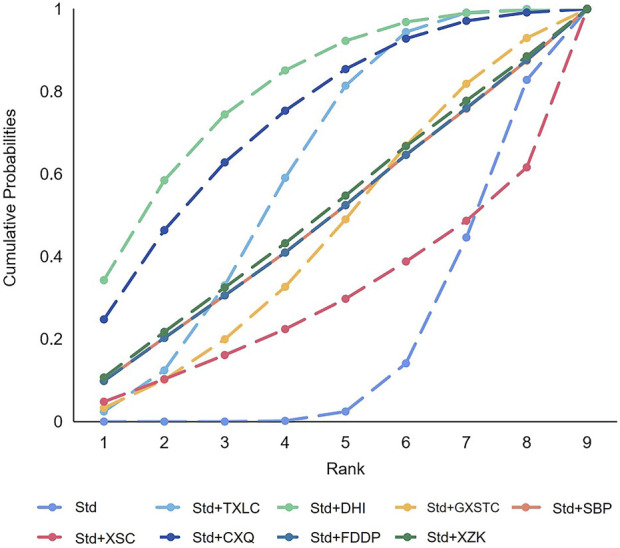
SUCRA curve with regard to reducing acute myocardial infarction after PCI. CXQ, Chuanxiongqin tablet; DHI, Danhong injection; FDDP, Fufang Danshen dripping pill; GXSTC, Guanxin Shutong capsule; QYDP, Qishen Yiqi dripping pill; SBP, Shexiang Baoxin pill; Std, standard treatment; TXLC, Tongxinluo capsule; XSC, Xiongshao capsule; SUCRA, surface under the cumulative ranking.

A total of 36 pairwise comparisons of AMI were generated by NMA (Figure 8). Only two pairwise direct comparisons were statistically significant. Compared with Std, DHI or TXLC reduced the risk of AMI by 89% (RR 0.11, 95% CI 0.00–0.69) and 73% (RR 0.27, 95% CI 0.10–0.64), respectively. As shown in Figure 9 and Supplementary Table S3 for the SUCRA of recurrence angina, DHI was most likely the best intervention. Certainty of evidence for all comparisons was very low to moderate. The details of evidence evaluation are available in Supplementary Table S6.

### 3.8 Secondary outcome: TLR

#### 3.8.1 Traditional pairwise meta-analysis

A traditional pairwise meta-analysis of the included study data was performed. There were 10 different interventions and nine pairwise comparisons generated. CXQ, FDDP, TXLC, or XSC was superior to Std (Supplementary Figure S6).

#### 3.8.2 Evidence network

In total, 15 studies including 10 medication strategies involving 1,839 patients reported TLR. The studies provided nine direct comparisons (Figure 3D). The number of studies that compared DHI with Std and the number of patients who received the treatment were 4 and 477, respectively, and these were the largest numbers compared with other direct comparisons.

#### 3.8.3 NMA and SUCRA

**FIGURE 10:**
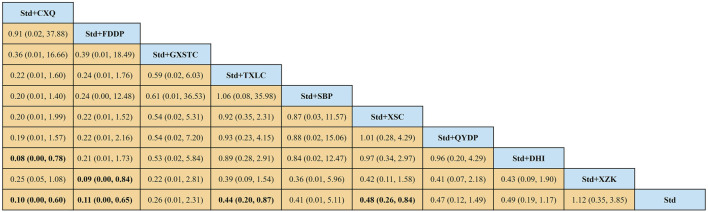
Summary of results of target lesion revascularization from network meta-analysis. CXQ, Chuanxiongqin tablet; DHI, Danhong injection; FDDP, Fufang Danshen dripping pill; GXSTC, Guanxin Shutong capsule; QYDP, Qishen Yiqi dripping pill; SBP, Shexiang Baoxin pill; Std, standard treatment; TXLC, Tongxinluo capsule; XSC, Xiongshao capsule; XZK, Xuezhikang capsule.

**FIGURE 11:**
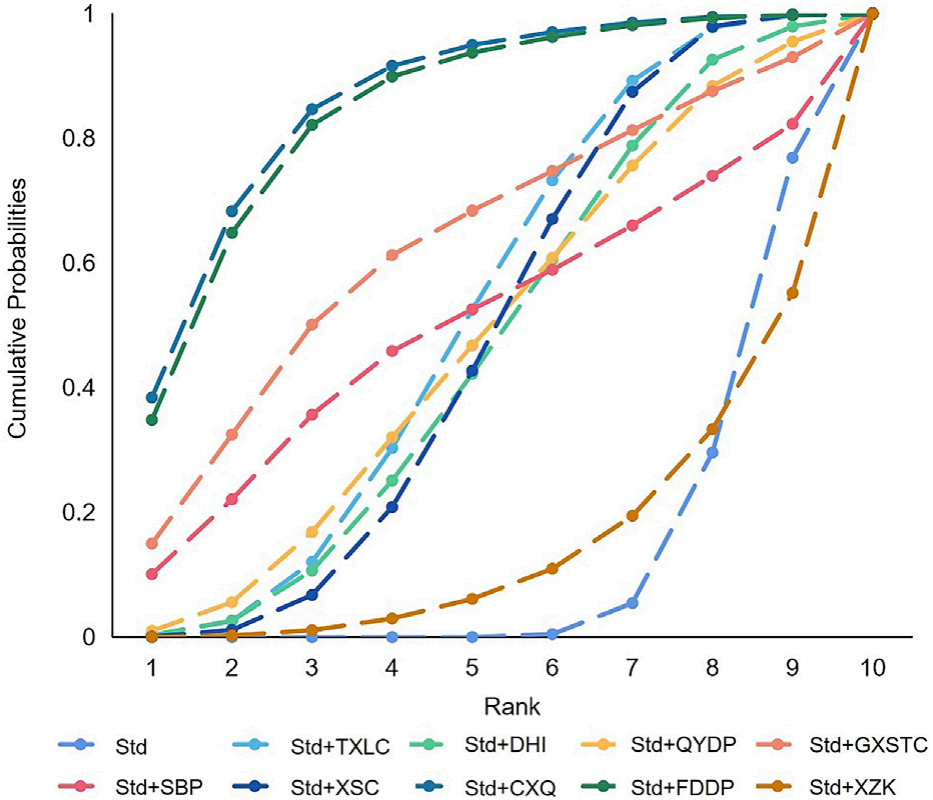
SUCRA curve with regard to reducing target lesion revascularization. CXQ, Chuanxiongqin tablet; DHI, Danhong injection; FDDP, Fufang Danshen dripping pill; GXSTC, Guanxin Shutong capsule; QYDP, Qishen Yiqi dripping pill; SBP, Shexiang Baoxin pill; Std, standard treatment; TXLC, Tongxinluo capsule; XSC, Xiongshao capsule; XZK, Xuezhikang capsule; SUCRA, surface under the cumulative ranking.

In total, 45 pairwise comparisons of TLR were generated by NMA (Figure 10). In addition to four pairwise direct comparisons, two pairwise indirect comparisons were also statistically significant. Compared with DHI, CXQ reduced the risk of TLR by 92% (RR 0.08, 95% CI 0.00–0.78). Compared with XZK, FDDP reduced the risk of TLR by 91% (RR 0.09, 95% CI 0.00–0.84). As shown in Figure 11 and Supplementary Table S3 for SUCRA, CXQ was most likely the best intervention. Certainty of evidence for all comparisons was very low to moderate. The details of evidence evaluation are available in Supplementary Table S7.

### 3.9 Subgroup and sensitivity analysis

The subgroup network meta-analysis for angiographic restenosis showed that QYDP performed better in studies involving ≥100 patients, while CXQ performed better in studies involving <100 patients (Supplementary Figures S7, S8). In sensitivity analysis, the results revealed that removing these studies with a high risk of bias had no discernible impact on the overall analysis (Supplementary Figures S9–S12).

### 3.10 Publication bias assessment

An assessment was conducted to determine the bias of different drugs regarding angiographic restenosis, recurrence angina, AMI after PCI, and TLR (Supplementary Figure S13). The included studies were represented by dots, with different interventions indicated by varying colors. The symmetry was acceptable.

## 4 Discussion

In this NMA, we reviewed, compared, and ranked the effectiveness of CPMs combined with Std in preventing restenosis after PCI. A total of 47 randomized controlled studies that enrolled a total of 5,077 patients assigned to 11 interventions were included in this study. Except XZK, CPMs including GXSTC, GXTLC, FDDP, CXQ, SBP, DHI, TXLC, QYDP, and XSC reduced the risk of angiographic restenosis. Among them, GXSTC showed the highest probability of being superior. For recurrent angina, FDDP was demonstrated to be the most effective intervention. In addition, DHI was more effective than other CPMs in preventing AMI after PCI. The network rank of the cumulative probability indicated that CXQ was the optimal treatment for TLR.

In fact, the previous meta-analyses have explored the effectiveness of TCM in preventing restenosis. Mao et al. (2015) conducted a systematic review of 16 RCTs involving 1,063 patients to evaluate the effectiveness of TXLC on patients with coronary heart disease after PCI. Similar to our findings, the results showed that TXLC reduced the risk of angiographic restenosis, recurrent angina, AMI, and revascularization by 84%, 76%, 68%, and 74%, respectively. Furthermore, TXLC exhibited a specific advantage in reducing the occurrence of adverse cardiovascular events without compromising safety on the 6-month course rather than the 3-month course, indicating that the effectiveness of TXLC might be influenced by different time courses (Hui et al., 2022). Our research results are also consistent with the meta-analysis study by Zheng et al. (2013) who reported that XSC has been beneficial in preventing restenosis after PCI in patients with coronary heart disease in certain aspects. Some other systematic reviews and meta-analyses reported that the combination treatment of TCM and Std showed promising results regarding preventing restenosis (Ren et al., 2008; Zheng et al., 2012; Chen et al., 2018; Wu et al., 2019). Compared with these studies, we not only analyzed the effectiveness of each CPM individually in preventing restenosis but also compared the effectiveness of different CPMs using a Bayesian NMA approach.

CPMs prevent restenosis through multiple targets and multiple ways (Shen, 2006). Clinical studies have demonstrated that FDDP (Tang and Zhang, 2015), DHI (Chen et al., 2015), XZK (Zhao et al., 2015), and TXLC (Sun and Lang, 2018) reduced the levels of blood lipids including cholesterol, low-density lipoprotein cholesterol, and triglyceride and suppressed inflammation by lowering hs-CRP, IL-6, TNF-α, and NF-κB. Furthermore, studies with animal models of arterial balloon injury were conducted to reveal the underlying mechanism. *Paeonia lactiflora* Pall. (Chi shao), one of the main components of TXLC and XSC, can reduce oxidative stress by targeting NADPH oxidase (Zhu and Zhu, 2004), inhibiting the expression of monocyte chemoattractant protein-1 mRNA (Zhu and Mou, 2008), and blocking type I collagen synthesis to prevent restenosis (Zhu and Zhu, 2004). FDDP also inhibits vascular smooth muscle cell proliferation, migration, and extracellular matrix synthesis and promotes the repair of damaged endothelial cells (Ma et al., 2006; Song, 2013), and TXLC can inhibit platelet aggregation (Huang et al., 2001), which are both the vital cause of restenosis.

Restenosis after PCI has remained an important clinical problem due to the higher mortality and poor prognosis once it occurs. Modern medicine generally focuses on the drug-release coatings of stents and balloons such as paclitaxel, sirolimus, everolimus, and rapamycin (Dibra et al., 2005; Stettler et al., 2008; Palmerini et al., 2015; Ahmad et al., 2022), which inhibit vascular smooth muscle cell proliferation and migration and prevent coronary artery restenosis by interrupting the cell cycle (Melnik et al., 2022). However, these agents also suppress the multiplication of vascular endothelial cells, resulting in incomplete endothelization and late thrombosis (Torii et al., 2020). Furthermore, a meta-analysis (Katsanos et al., 2018) reported that paclitaxel-coated balloons and stents increased the risk of death following application in the femoropopliteal artery of the lower limbs. Subsequently, the Federal Drug Administration alerted healthcare providers to continue closely monitoring this problem.

Another way to prevent restenosis is that modern medicine concerned with is the materials of opening the stenosed or occluded coronary arteries. Trials that compared permanent drug-eluting stent implantation with drug-coated balloon dilation (Cortese et al., 2023; 2020; Jeger et al., 2020) or bioresorbable drug-eluting stent implantation (Saito et al., 2014; Zong et al., 2022) have yielded neutral or somewhat exciting results. Nevertheless, drug-coated balloons are only suitable for a limited population and bioresorbable drug-eluting stents face technical challenges, leading to limited clinical application. To the best of our knowledge, there have been few studies on oral contemporary Western medicine to prevent restenosis. The randomized controlled trial named “OPTION” (indobufen or aspirin on top of clopidogrel after coronary drug-eluting stent implantation) (Wu et al., 2023) still failed to elucidate the antiplatelet mechanism. Therefore, considering new therapeutic strategies such as TCM may be feasible based on the complex mechanism involved in restenosis.

Some reasons may affect the extrapolation of our results. Clinical predictors of coronary restenosis include older age, diabetes mellitus, female sex, and higher body mass index (Giustino et al., 2022). However, more than 95% of the patients included in this study were ≤65 years of age, and individual-level data on gender, body mass index, and history of diabetes are not available. The efficacy of Chinese patent medicine in preventing restenosis in these populations is unclear. Future additional randomized controlled trials of different populations are needed.

In summary, this NMA provided a comprehensive picture of the likelihood of a range of CPMs to prevent coronary restenosis. We also reported the rank probability for all 11 treatment strategies including standard treatment using the Bayesian approach. The Bayesian approach has significant advantages over classical frequentist statistical approach for presenting evidence to decision-makers. Therefore, this study provided a reference recommendation for clinicians on further reducing the risk of restenosis for patients receiving PCI and which CAP should be chosen.

Our study also had several limitations. First, included studies did not clearly describe randomization procedures or whether allocation concealment and blinding occurred, leading to “some concerns” about quality of evidence of most studies, which may affect the analysis results. The treatment effects of CPMs must be evaluated by rigorous RCT design in the future. Second, lacking direct head-to-head trials led to our finding of low or very low certainty of evidence. Third, the most effective drugs for different outcomes were inconsistent, confusing clinicians when prescribing them. Based on the above limitation, treatment rank may have a substantial degree of imprecision, and the results in terms of treatment rank should be interpreted with caution although we performed the Bayesian approach.

## 5 Conclusion

This NMA indicated that addition of CPMs to standard treatment may prevent restenosis in patients with coronary heart disease (CHD) after PCI. GXSTC, FDDP, DHI, and CXQ combined with Std were more effective for angiographic restenosis, recurrence angina, AMI after PCI, and TLR, respectively. However, interpreting these results should be cautious due to the poor quality of original research and low grade of evidence with this study.

## Data Availability

The raw data supporting the conclusion of this article will be made available by the authors, without undue reservation.

## Abbreviations

ACS, acute coronary syndrome; AMI, acute myocardial infarction; CI, confidence interval; CHD, coronary heart disease; CPMs, Chinese patent medicines; CXQ, Chuanxiongqin tablet; DHI, Danhong injection; FDDP, Fufang Danshen dripping pill; GXSTC, Guanxin Shutong capsule; GXTLC, Guanxin Tongluo capsule; NMA, network meta-analysis; PCI, percutaneous coronary intervention; RCT, randomized controlled trial; RR, relative risk; SBP, Shexiang Baoxin pill; SUCRA, surface under the cumulative ranking; TCM, traditional Chinese medicine; TLR, target lesion revascularization; Std, standard treatment; TXLC, Tongxinluo capsule; XSC, Xiongshao capsule; QYDP, Qishen Yiqi dripping pill; XZK, Xuezhikang capsule.
