# Supplementary material for: Comparative efficacy of different Chinese patent medicines in preventing restenosis after percutaneous coronary intervention: a systematic review and Bayesian network meta-analysis of randomized clinical trials
Source: Front Pharmacol. 2024 Jan 5;14:1265766. doi: 10.3389/fphar.2023.1265766 (PMC10796822; doi:10.3389/fphar.2023.1265766)
Supplement: Supplementary file 2 [file DataSheet1.docx]

Supplementary Material

Supplement to:

**Comparative efficacy of different Chinese patent medicines in preventing restenosis after percutaneous coronary intervention: A systematic review and Bayesian network meta-analysis of randomized clinical trials**

Jiasai Fan^1†^, Tianli Li^2†^, Fenglan Pu^3^^†^, Nan Guo^4†^, Jing Wang^1^, Yuqian Gao^1^, Hongbing Zhao^1^, Xian Wang^1,5*^, Haiyan Zhu^1*^

# Standard evaluation of Chinese patent medicines

## Table 1: Detail information of Chinese patent medicines

| Drug name | Source | Compositions | Usage and dosage (Medicine instruction) | Quality control reported? | Chemical analysis reported? |
| --- | --- | --- | --- | --- | --- |
| Tongxinluo capsule | Shijiazhuang Yiling Pharmaceutical Co., Ltd. | *Panax ginseng* C.A.Mey. [Araliaceae; *Panax ginseng* root], *Scorpio** [Buthidae; *Buthus martensii* whole body], *Hirudo** [Hirudinidae; *Hirudo nipponica* whole body], *Eupolyphaga steleophage** [Periplanetidae; *Eupolyphaga* *sinensis* whole body (female)], *Scolopendra** [Scolopendridae; *Scolopendra subspinipes mutilans* whole body], *Cicadae periostracum** [Cicadidae; *Cryptotympana pustulata* nymph shell], *Paeonia lactiflora* Pall. [Paeoniaceae; *Paeonia lactiflora* root] (chi shao), *Borneolum syntheticum** [Artificially synthesized], *Santalum album* L. [Santalaceae; *Santalum album* wood], *Dalbergia odorifera* T.C.Chen [Fabaceae; *Dalbergia odorifera* wood], *Boswellia sacra* Flück. [Burseraceae; *Boswellia sacra* oleo-gum-resin], *Ziziphus jujuba* Mill. [Rhamnaceae; *Ziziphus jujuba* seed] | 2-4 capsules (0.26g/ capsule), tid, po | Y-Prepared according to NMPA: Z19980015 | N |
| Danhong injection | Shandong Danhong Pharmaceutical Co., Ltd or Buchang Pharmaceutical Co., Ltd. | *Salvia miltiorrhiza* Bunge [Lamiaceae; *Salvia miltiorrhiza* rhizome et root], *Carthamus tinctorius* L. [Asteraceae; *Carthamus tinctorius* flower] | 20-40ml, qd or bid, ivgtt | Y-Prepared according to NMPA: Z20026866 | N |
| Qishen Yiqi dripping pill | Tasly Pharmaceutical Group Co., Ltd. | *Astragalus mongholicus* Bunge [Fabaceae; *Astragalus mongholicus* root], *Salvia miltiorrhiza* Bunge [Lamiaceae; *Salvia miltiorrhiza* rhizome et root], *Panax notoginseng* (Burkill) F.H.Chen [Araliaceae; *Panax notoginseng* root], *Dalbergia odorifera* T.C.Chen [Fabaceae; *Dalbergia odorifera* wood] | 1 bag (0.50g or 0.52g/bag), tid, po | Y-Prepared according to NMPA: Z20030139 and Z20113048 | N |
| Guanxin Shutong capsule | Shaanxi Buchang Pharmaceutical Co., Ltd. | *Salvia miltiorrhiza* Bunge [Lamiaceae; *Salvia miltiorrhiza* rhizome et root], *Syzygium aromaticum* (L.) Merr. & L.M.Perry [Myrtaceae; *Syzygium aromaticum* flower], *Borneolum syntheticum** [Artificially synthesized], *Bambusa textilis* McClure [Poaceae; dried mass of secretion from *Bambusa textilis* stem], *Choerospondias axillaris* (Roxb.) B.L.Burtt & A.W.Hill [Anacardiaceae; *Choerospondias axillaris* fruit] | 3 capsules (0.3g/capsule), tid, po | Y-Prepared according to NMPA: Z20020055 | N |
| Shexiang Baoxin pill | Shanghai Hehuang Pharmaceutical Co., Ltd | *Moschus** [Cervidae; dried secretions from mature *Moschus berezovskii* (male) sachets], *Panax ginseng* C.A.Mey. [Araliaceae; *Panax ginseng* root], *Bovis calculus** [Bovidae; *Bos taurus domesticus* gallstone], *Cinnamomum verum* J.Presl [Lauraceae; *Cinnamomum verum* bark], *Liquidambar orientalis* Mill. [Altingiaceae; purified balsam from *Liquidambar orientalis* trunk], *Bufonis Venenum** [Bufonidae; dried secretions from *Bufo bufo gargarizans*], *Borneolum syntheticum** [Artificially synthesized] | 1-2 pills (22.5mg/pill), tid, po | Y-Prepared according to NMPA: Z31020068 | N |
| Xiongshao capsule | Jiangsu Nhwa Pharmaceutical Co., Ltd or Suzhou Institute of Traditional Chinese Medicine or Xiyuan Hospital | *Conioselinum anthriscoides* (H.Boissieu) Pimenov & Kljuykov [Apiaceae; *Conioselinum anthriscoides* rhizome et root], *Paeonia lactiflora* Pall. [Paeoniaceae; *Paeonia lactiflora* root] (chi shao) | N | N | N |
| Chuanxiongqin tablet | Beijing Yanjing Pharmaceutical Co., Ltd# | *Ligustrazine* | 50-100mg (50mg/tablet), tid, po# | Y-Prepared according to NMPA: H11021964# | N |
| Guanxin Tongluo capsule | N | *Astragalus mongholicus* Bunge [Fabaceae; *Astragalus mongholicus* root], *Salvia miltiorrhiza* Bunge [Lamiaceae; *Salvia miltiorrhiza* rhizome et root], *Carthamus tinctorius* L. [Asteraceae, *Carthamus tinctorius* flower], *Trichosanthes kirilowii* Maxim. [Cucurbitaceae; *Trichosanthes kirilowii* seed], *Ophiopogon japonicus* (Thunb.) Ker Gawl. [Asparagaceae; *Ophiopogon japonicus* root tuber], *Glycyrrhiza glabra* L. [Fabaceae; *Glycyrrhiza glabra* rhizome et root] | N | N | N |
| Fufang Danshen dripping pill | Tasly Pharmaceutical Group Co., Ltd. | *Salvia miltiorrhiza* Bunge [Lamiaceae; *Salvia miltiorrhiza* rhizome et root], *Panax notoginseng* (Burkill) F.H.Chen [Araliaceae; *Panax notoginseng* root], *Borneolum syntheticum** [Artificially synthesized] | 10 pills (27mg/pill), tid, po | Y-Prepared according to NMPA: Z10950111 | N |
| Xuezhikang capsule | Beijing Peking University WBL Biotech Co., Ltd | An extract from red yeast rice | 2 capsules (0.3g/capsule), bid, po | Y-Prepared according to NMPA: Z10950029 | N |

## *: non-botanical drug, the names refer to the Chinese pharmacopoeia 2020.

## Table 2: Extract and extraction process description of Chinese patent medicines

| Drug name | Extract and extraction process description |
| --- | --- |
| Tongxinluo capsule | N |
| Danhong injection | Mix 250 g *Carthamus tinctorius* L., 750 g *Salvia miltiorrhiza* Bunge, and 7 g Sodium Chloride for Injection to make a 1000 mL solution. Before that, *Salvia miltiorrhiza* Bunge is soaked twice with dilute ethanol for 1 hour each time, filter, and the filtrate is stored for future use. The residue is mixed with *Carthamus tinctorius* L., soak twice with water for 1 hour each time, filter, and the filtrate is combined and concentrated to an ointment with a relative density of 1.10 to 1.20 (65 °C), sodium chloride for injection is added to reach isotonicity, pH is adjusted to 6 to 7, filter, and refrigerate for 24 hours. Injection water is added to the specified amount. Then filter, fill in a container and seal, sterilize, and the Danhong injection is ready. |
| Qishen Yiqi dripping pill | *Salvia miltiorrhiza* Bunge and *Panax notoginseng* (Burkill) F.H.Chen are decocted twice with water for 2 hours each time. The filtrate is filtered and concentrated to a relative density of 1.13 to 1.23 (80 ℃). Ethanol is added to achieve a 70% ethanol content. The filtrate is then allowed to stand and filter, ethanol is recovered and concentrated into a thick ointment. *Astragalus mongholicus* Bunge is boiled twice with water for 2 hours for the first time and 1 hour for the second time. After filtration, the filtrate was concentrated to a relative density of 1.05 to 1.20 (75 ℃). Ethanol is added to achieve a 60% ethanol content. The filtrate is then allowed to stand, filter, and ethanol is recovered to a relative density of 1.18 to 1.30 (60 ℃). Ethanol is added to achieve an ethanol content of 80%. After filtration, ethanol is recovered and concentrated into a thick ointment. Combine the two thick ointments mentioned above, add an appropriate amount of polyethylene glycol 6000, heat and melt, add *Dalbergia odorifera* T.C.Chen oil, mix well, and make 1050g drop pills, then the Danhong injection is ready. |
| Guanxin Shutong capsule | Take 120g *Choerospondias axillaris* (Roxb.) B.L.Burtt & A.W.Hill and crush it into fine powder, then divide it evenly into two parts for later use. Crush *Bambusa textilis* McClure into fine powder, set aside, and crush the remaining *Choerospondias axillaris* (Roxb.) B.L.Burtt & A.W.Hill into the coarsest powder. Use 70% ethanol as a solvent for percolation, collect the percolation solution, recover ethanol, and concentrate it to a thick ointment with a relative density of 1.30 to 1.35 (50 ℃). Add a portion of *Choerospondias axillaris* (Roxb.) B.L.Burtt & A.W.Hill fine powder, mix well, dry, and crush it into fine powder, set aside. Extract *Salvia miltiorrhiza* Bunge three times, heat and reflux with ethanol for 1.5 hours for the first time, filter, recover ethanol from the filtrate, and concentrate it to a thick ointment with a relative density of 1.30 to 1.35 (55 to 60 ℃) for later use. Heat and reflux with 50% ethanol for 1.5 hours for the second time, filter, and reserve the filtrate. Boil with water for 2 hours for the third time, filter, merge the filtrate with the filtrate extracted from the second time, recover ethanol, and concentrate it to a thick ointment with a relative density of 1.30 to 1.35 (55to 60 ℃). Combine it with the thick ointment extracted from the first time, mix well, and concentrate it to a thick ointment with a relative density of 1.30 to 1.35 (55 to 60 ℃). Add another portion of *Choerospondias axillaris* (Roxb.) B.L.Burtt & A.W.Hill powder, mix well, dry, and crush it into a fine powder. Extract volatile oil from *Syzygium aromaticum* (L.) Merr. & L.M.Perry by steam distillation, spray the volatile oil evenly into 15g of fine powder of *Bambusa textilis* McClure, mix well, and seal tightly. Mix *Borneolum syntheticum* with other fine powders of *Bambusa textilis* McClure, crush it into fine powder, mix with each of the above fine powders, put it into capsules, and make 1000 capsules, then Guanxin Shutong capsule is ready. |
| Shexiang Baoxin pill | *Moschus*, *Panax ginseng* C.A.Mey., *Bovis calculus, Cinnamomum verum* J. Presl, *Bufonis Venenum*, *Borneolum syntheticum* are crushed together into fine powder. Take *Liquidambar orientalis* Mill. and add proper amount of Baijiu to make pills, dry, then Shexiang Baoxin pill is ready. |
| Xiongshao capsule | N |
| Chuanxiongqin tablet | N |
| Guanxin Tongluo capsule | N |
| Fufang Danshen dripping pill | Grind the *Borneolum syntheticum* finely. *Salvia miltiorrhiza* Bunge and *Panax notoginseng* (Burkill) F.H.Chen are boiled in water, the decoction is filtered, the filtrate is concentrated, ethanol is added, left to stand, the supernatant is taken, ethanol is recovered, and concentrated into a thick ointment for later use. Take an appropriate amount of polyethylene glycol, heat to melt, add the above thick ointment and fine borneol powder, mix well, and drop into cooled liquid paraffin to make drop pills, then Fufang Danshen dripping pill is ready. |
| Xuezhikang capsule | Add a certain concentration of ethanol to the remaining red yeast rice for reflux extraction twice, filter, merge the filtrate, recover ethanol, concentrate to an appropriate amount, spray into red yeast rice powder, make particles, and put them into 1000 capsules, then Xuezhikang capsule is ready. |

# Table 3: The probability ranks of Chinese patent medicine with different outcomes

| Intervention | Angiographic restenosis | | Recurrence angina | | AMI | | TLR | |
| --- | --- | --- | --- | --- | --- | --- | --- | --- |
|  | SUCRA (%) | Rank | SUCRA (%) | Rank | SUCRA (%) | Rank | SUCRA (%) | Rank |
| Std | 4.45 | 11 | 0.30 | 9 | 18.03 | 9 | 12.49 | 10 |
| Std+TXLC | 40.27 | 7 | 75.42 | 2 | 60.23 | 3 | 50.86 | 4 |
| Std+DHI | 59.52 | 6 | 54.26 | 5 | 80.03 | 1 | 45.64 | 8 |
| Std+QYDP | 35.78 | 8 | 37.43 | 6 | - | - | 46.95 | 7 |
| Std+GXSTC | 80.54 | 1 | 21.18 | 8 | 44.61 | 7 | 62.65 | 3 |
| Std+SBP | 64.41 | 5 | 32.42 | 7 | 47.74 | 6 | 49.72 | 5 |
| Std+XSC | 28.31 | 9 | 67.64 | 4 | 29.07 | 8 | 47.09 | 6 |
| Std+CXQ | 70.88 | 4 | 72.22 | 3 | 72.98 | 2 | 85.87 | 1 |
| Std+GXTLC | 77.96 | 2 | - | - | - | - | - | - |
| Std+FDDP | 76.49 | 3 | 89.13 | 1 | 47.81 | 5 | 84.33 | 2 |
| Std+XZK | 11.39 | 10 | - | - | 49.50 | 4 | 14.38 | 9 |

AMI: acute myocardial infarction; TRL: target lesion revascularization; CXQ: Chuanxiongqin tablet; DHI: Danhong injection; FDDP: Fufang Danshen dripping pill; GXSTC: Guanxin Shutong capsule; GXTLC: Guanxin Tongluo capsule; QYDP: Qishen Yiqi dripping pill; SBP: Shexiang Baoxin pill; Std: standard treatment; TXLC: Tongxinluo capsule; XSC: Xiongshao capsule; XZK: Xuezhikang capsule. SUCRA: surface under the cumulative ranking curves.

# GRADE assessments

## Table 4: GRADE assessments of angiographic restenosis

| Comparison | Direct Estimate | Certainty | Indirect Estimate | Certainty | Network Estimate | Certainty |
| --- | --- | --- | --- | --- | --- | --- |
| GXSTC + Std vs GXTLC + Std | — | — | 1.00 (0.31, 3.57) | Low | 1.00 (0.31, 3.57) | Low |
| GXSTC + Std vs FDDP + Std | — | — | 0.97 (0.31, 3.45) | Low | 0.97 (0.31, 3.45) | Low |
| GXSTC + Std vs CXQ + Std | — | — | 0.85 (0.3, 2.43) | Low | 0.85 (0.3, 2.43) | Low |
| GXSTC + Std vs SBP + Std | — | — | 0.74 (0.32, 1.59) | Low | 0.74 (0.32, 1.59) | Low |
| GXSTC + Std vs DHI + Std | — | — | 0.70 (0.28, 1.65) | Low | 0.70 (0.28, 1.65) | Low |
| GXSTC + Std vs TXLC + Std | — | — | 0.55 (0.25, 1.12) | Moderate | 0.55 (0.25, 1.12) | Moderate |
| GXSTC + Std vs QYDP + Std | — | — | 0.51 (0.21, 1.16) | Low | 0.51 (0.21, 1.16) | Low |
| GXSTC + Std vs XSC + Std | — | — | 0.47 (0.21, 0.99) | Moderate | 0.47 (0.21, 0.99) | Moderate |
| GXSTC + Std vs XZK + Std | — | — | 0.25 (0.06, 0.95) | Very low | 0.25 (0.06, 0.95) | Very low |
| GXSTC + Std vs Std | 0.24 (0.11, 0.45) | Moderate | — | — | 0.24 (0.11, 0.45) | Moderate |
| GXTLC + Std vs FDDP + Std | — | — | 0.97 (0.24, 3.89) | Low | 0.97 (0.24, 3.89) | Low |
| GXTLC + Std vs CXQ + Std | — | — | 0.84 (0.23, 2.83) | Low | 0.84 (0.23, 2.83) | Low |
| GXTLC + Std vs SBP + Std | — | — | 0.74 (0.23, 1.94) | Low | 0.74 (0.23, 1.94) | Low |
| GXTLC + Std vs DHI + Std | — | — | 0.7 (0.21, 1.98) | Low | 0.7 (0.21, 1.98) | Low |
| GXTLC + Std vs TXLC + Std | — | — | 0.55 (0.18, 1.38) | Low | 0.55 (0.18, 1.38) | Low |
| GXTLC + Std vs QYDP + Std | — | — | 0.51 (0.16, 1.40) | Low | 0.51 (0.16, 1.40) | Low |
| GXTLC + Std vs XSC + Std | — | — | 0.47 (0.15, 1.21) | Very low | 0.47 (0.15, 1.21) | Very low |
| GXTLC + Std vs XZK + Std | — | — | 0.25 (0.05, 1.08) | Low | 0.25 (0.05, 1.08) | Low |
| GXTLC + Std vs Std | 0.23 (0.08, 0.56) | Moderate | — | — | 0.23 (0.08, 0.56) | Moderate |
| FDDP + Std vs CXQ + Std | — | — | 0.87 (0.24, 2.89) | Low | 0.87 (0.24, 2.89) | Low |
| FDDP + Std vs SBP + Std | — | — | 0.76 (0.24, 1.98) | Low | 0.76 (0.24, 1.98) | Low |
| FDDP + Std vs DHI + Std | — | — | 0.72 (0.22, 2.02) | Low | 0.72 (0.22, 2.02) | Low |
| FDDP + Std vs TXLC + Std | — | — | 0.57 (0.19, 1.40) | Low | 0.57 (0.19, 1.40) | Low |
| FDDP + Std vs QYDP + Std | — | — | 0.53 (0.16, 1.43) | Moderate | 0.53 (0.16, 1.43) | Moderate |
| FDDP + Std vs XSC + Std | — | — | 0.49 (0.16, 1.23) | Moderate | 0.49 (0.16, 1.23) | Moderate |
| FDDP + Std vs XZK + Std | — | — | 0.26 (0.05, 1.09) | Low | 0.26 (0.05, 1.09) | Low |
| FDDP + Std vs Std | 0.24 (0.08, 0.57) | Moderate | — | — | 0.24 (0.08, 0.57) | Moderate |
| CXQ + Std vs SBP + Std | — | — | 0.87 (0.35, 1.95) | Low | 0.87 (0.35, 1.95) | Low |
| CXQ + Std vs DHI + Std | — | — | 0.83 (0.31, 2.02) | Very low | 0.83 (0.31, 2.02) | Very low |
| CXQ + Std vs TXLC + Std | — | — | 0.65 (0.28, 1.37) | Moderate | 0.65 (0.28, 1.37) | Moderate |
| CXQ + Std vs QYDP + Std | — | — | 0.61 (0.24, 1.42) | Low | 0.61 (0.24, 1.42) | Low |
| CXQ + Std vs XSC + Std | — | — | 0.56 (0.23, 1.21) | Low | 0.56 (0.23, 1.21) | Low |
| CXQ + Std vs XZK + Std | — | — | 0.30 (0.07, 1.14) | Low | 0.30 (0.07, 1.14) | Low |
| CXQ + Std vs Std | 0.28 (0.12, 0.56) | Moderate | — | — | 0.28 (0.12, 0.56) | Moderate |
| SBP + Std vs DHI + Std | — | — | 0.95 (0.50, 1.85) | Low | 0.95 (0.50, 1.85) | Low |
| SBP + Std vs TXLC + Std | — | — | 0.75 (0.47, 1.16) | Moderate | 0.75 (0.47, 1.16) | Moderate |
| SBP + Std vs QYDP + Std | — | — | 0.69 (0.38, 1.28) | Low | 0.69 (0.38, 1.28) | Low |
| SBP + Std vs XSC + Std | — | — | 0.64 (0.39, 1.05) | Low | 0.64 (0.39, 1.05) | Low |
| SBP + Std vs XZK + Std | — | — | 0.34 (0.10, 1.13) | Low | 0.34 (0.10, 1.13) | Low |
| SBP + Std vs Std | 0.32 (0.21, 0.46) | Moderate | — | — | 0.32 (0.21, 0.46) | Moderate |
| DHI + Std vs TXLC + Std | — | — | 0.79 (0.43, 1.38) | Low | 0.79 (0.43, 1.38) | Low |
| DHI + Std vs QYDP + Std | — | — | 0.73 (0.36, 1.48) | Low | 0.73 (0.36, 1.48) | Low |
| DHI + Std vs XSC + Std | — | — | 0.67 (0.36, 1.24) | Low | 0.67 (0.36, 1.24) | Low |
| DHI + Std vs XZK + Std | — | — | 0.36 (0.10, 1.26) | Low | 0.36 (0.10, 1.26) | Low |
| DHI + Std vs Std | 0.34 (0.19, 0.55) | Moderate | — | — | 0.34 (0.19, 0.55) | Moderate |
| TXLC + Std vs QYDP + Std | — | — | 0.93 (0.55, 1.60) | Moderate | 0.93 (0.55, 1.60) | Moderate |
| TXLC + Std vs XSC + Std | — | — | 0.85 (0.57, 1.29) | Low | 0.85 (0.57, 1.29) | Low |
| TXLC + Std vs XZK + Std | — | — | 0.46 (0.14, 1.46) | Low | 0.46 (0.14, 1.46) | Low |
| TXLC + Std vs Std | 0.42 (0.33, 0.54) | Moderate | — | — | 0.42 (0.33, 0.54) | Moderate |
| QYDP + Std vs XSC + Std | — | — | 0.92 (0.51, 1.63) | Low | 0.92 (0.51, 1.63) | Low |
| QYDP + Std vs XZK + Std | — | — | 0.49 (0.14, 1.69) | Low | 0.49 (0.14, 1.69) | Low |
| QYDP + Std vs Std | 0.46 (0.28, 0.72) | Moderate | — | — | 0.46 (0.28, 0.72) | Moderate |
| XSC + Std vs XZK + Std | — | — | 0.54 (0.16, 1.76) | Low | 0.54 (0.16, 1.76) | Low |
| XSC + Std vs Std | 0.50 (0.35, 0.68) | Moderate | — | — | 0.50 (0.35, 0.68) | Moderate |
| XZK + Std vs Std | 0.92 (0.30, 2.90) | Moderate | — | — | 0.92 (0.30, 2.90) | Moderate |

## Table 5: GRADE assessments of recurrence angina

| Comparison | Direct Estimate | Certainty | Indirect Estimate | Certainty | Network Estimate | Certainty |
| --- | --- | --- | --- | --- | --- | --- |
| FDDP + Std vs TXLC + Std | — | — | 0.60 (0.13, 1.87) | Moderate | 0.60 (0.13, 1.87) | Moderate |
| FDDP + Std vs XSC + Std | — | — | 0.62 (0.11, 2.94) | Low | 0.62 (0.11, 2.94) | Low |
| FDDP + Std vs CXQ + Std | — | — | 0.55 (0.12, 1.74) | Low | 0.55 (0.12, 1.74) | Low |
| FDDP + Std vs DHI + Std | — | — | 0.45 (0.09, 1.60) | Low | 0.45 (0.09, 1.60) | Low |
| FDDP + Std vs QYDP + Std | — | — | 0.35 (0.07, 1.31) | Very low | 0.35 (0.07, 1.31) | Very low |
| FDDP + Std vs SBP + Std | — | — | 0.33 (0.07, 1.09) | Low | 0.33 (0.07, 1.09) | Low |
| FDDP + Std vs GXSTC + Std | — | — | 0.28 (0.06, 0.89) | Low | 0.28 (0.06, 0.89) | Low |
| FDDP + Std vs Std | 0.17 (0.04, 0.51) | Moderate | — | — | 0.17 (0.04, 0.51) | Moderate |
| TXLC + Std vs XSC + Std | — | — | 1.03 (0.41, 3.22) | Low | 1.03 (0.41, 3.22) | Low |
| TXLC + Std vs CXQ + Std | — | — | 0.91 (0.57, 1.46) | Low | 0.91 (0.57, 1.46) | Low |
| TXLC + Std vs DHI + Std | — | — | 0.75 (0.40, 1.51) | Very low | 0.75 (0.40, 1.51) | Very low |
| TXLC + Std vs QYDP + Std | — | — | 0.58 (0.29, 1.28) | Low | 0.58 (0.29, 1.28) | Low |
| TXLC + Std vs SBP + Std | — | — | 0.56 (0.33, 0.95) | Low | 0.56 (0.33, 0.95) | Low |
| TXLC + Std vs GXSTC + Std | — | — | 0.46 (0.29, 0.76) | Low | 0.46 (0.29, 0.76) | Low |
| TXLC + Std vs Std | 0.29 (0.21, 0.38) | Moderate | — | — | 0.29 (0.21, 0.38) | Moderate |
| XSC + Std vs CXQ + Std | — | — | 0.88 (0.28, 2.28) | Low | 0.88 (0.28, 2.28) | Low |
| XSC + Std vs DHI + Std | — | — | 0.73 (0.21, 2.14) | Moderate | 0.73 (0.21, 2.14) | Moderate |
| XSC + Std vs QYDP + Std | — | — | 0.57 (0.16, 1.76) | Low | 0.57 (0.16, 1.76) | Low |
| XSC + Std vs SBP + Std |  |  | 0.54 (0.17, 1.43) | Low | 0.54 (0.17, 1.43) | Low |
| XSC + Std vs GXSTC + Std | — | — | 0.45 (0.14, 1.17) | Low | 0.45 (0.14, 1.17) | Low |
| XSC + Std vs Std | 0.28 (0.09, 0.66) | Moderate | — | — | 0.28 (0.09, 0.66) | Moderate |
| CXQ + Std vs DHI + Std | — | — | 0.83 (0.42, 1.70) | Low | 0.83 (0.42, 1.70) | Low |
| CXQ + Std vs QYDP + Std | — | — | 0.65 (0.30, 1.44) | Low | 0.65 (0.30, 1.44) | Low |
| CXQ + Std vs SBP + Std | — | — | 0.61 (0.35, 1.07) | Low | 0.61 (0.35, 1.07) | Low |
| CXQ + Std vs GXSTC + Std | — | — | 0.51 (0.30, 0.86) | Low | 0.51 (0.30, 0.86) | Low |
| CXQ + Std vs Std | 0.32 (0.22, 0.45) | Moderate | — | — | 0.32 (0.22, 0.45) | Moderate |
| DHI + Std vs QYDP + Std | — | — | 0.78 (0.32, 1.94) | Low | 0.78 (0.32, 1.94) | Low |
| DHI + Std vs SBP + Std | — | — | 0.74 (0.35, 1.51) | Low | 0.74 (0.35, 1.51) | Low |
| DHI + Std vs GXSTC + Std | — | — | 0.61 (0.30, 1.22) | Low | 0.61 (0.30, 1.22) | Low |
| DHI + Std vs Std | 0.38 (0.20, 0.67) | Moderate | — |  | 0.38 (0.20, 0.67) | Moderate |
| QYDP + Std vs SBP + Std | — | — | 0.95 (0.41, 2.09) | Moderate | 0.95 (0.41, 2.09) | Moderate |
| QYDP + Std vs GXSTC + Std | — | — | 0.79 (0.35, 1.69) | Low | 0.79 (0.35, 1.69) | Low |
| QYDP + Std vs Std | 0.49 (0.24, 0.94) | Moderate | — | — | 0.49 (0.24, 0.94) | Moderate |
| SBP + Std vs GXSTC + Std |  |  | 0.83 (0.47, 1.48) | Low | 0.83 (0.47, 1.48) | Low |
| SBP + Std vs Std | 0.52 (0.33, 0.79) | Moderate | — | — | 0.52 (0.33, 0.79) | Moderate |
| GXSTC + Std vs Std | 0.62 (0.42, 0.90) | Moderate | — | — | 0.62 (0.42, 0.90) | Moderate |

## Table 6: GRADE assessments of AMI

| Comparison | Direct Estimate | Certainty | Indirect Estimate | Certainty | Network Estimate | Certainty |
| --- | --- | --- | --- | --- | --- | --- |
| DHI + Std vs CXQ + Std | — | — | 0.71 (0.02, 29.92) | Low | 0.71 (0.02, 29.92) | Low |
| DHI + Std vs TXLC + Std | — | — | 0.39 (0.01, 3.35) | Low | 0.39 (0.01, 3.35) | Low |
| DHI + Std vs XZK + Std | — | — | 0.26 (0.00, 13.64) | Low | 0.26 (0.00, 13.64) | Low |
| DHI + Std vs FDDP + Std | — | — | 0.24 (0.00, 12.99) | Low | 0.24 (0.00, 12.99) | Low |
| DHI + Std vs SBP + Std | — | — | 0.24 (0.00, 12.28) | Low | 0.24 (0.00, 12.28) | Low |
| DHI + Std vs GXSTC + Std | — | — | 0.22 (0.01, 3.81) | Low | 0.22 (0.01, 3.81) | Low |
| DHI + Std vs XSC + Std | — | — | 0.10 (0.00, 6.06) | Very low | 0.10 (0.00, 6.06) | Very low |
| DHI + Std vs Std | 0.11 (0.00, 0.69) | Moderate | — | — | 0.11 (0.00, 0.69) | Moderate |
| CXQ + Std vs TXLC + Std | — | — | 0.54 (0.02, 4.92) | Low | 0.54 (0.02, 4.92) | Low |
| CXQ + Std vs XZK + Std | — | — | 0.37 (0.01, 19.70) | Low | 0.37 (0.01, 19.70) | Low |
| CXQ + Std vs FDDP + Std | — | — | 0.35 (0.01, 18.72) | Very low | 0.35 (0.01, 18.72) | Very low |
| CXQ + Std vs SBP + Std | — | — | 0.34 (0.01, 17.98) | Low | 0.34 (0.01, 17.98) | Low |
| CXQ + Std vs GXSTC + Std | — | — | 0.32 (0.01, 5.57) | Low | 0.32 (0.01, 5.57) | Low |
| CXQ + Std vs XSC + Std | — | — | 0.14 (0.00, 8.87) | Low | 0.14 (0.00, 8.87) | Low |
| CXQ + Std vs Std | 0.15 (0.01, 1.04) | Moderate | — | — | 0.15 (0.01, 1.04) | Moderate |
| TXLC + Std vs XZK + Std | — | — | 0.70 (0.05, 23.95) | Very low | 0.70 (0.05, 23.95) | Very low |
| TXLC + Std vs FDDP + Std | — | — | 0.66 (0.04, 23.23) | Moderate | 0.66 (0.04, 23.23) | Moderate |
| TXLC + Std vs SBP + Std | — | — | 0.65 (0.04, 22.35) | Low | 0.65 (0.04, 22.35) | Low |
| TXLC + Std vs GXSTC + Std |  |  | 0.59 (0.09, 5.51) | Low | 0.59 (0.09, 5.51) | Low |
| TXLC + Std vs XSC + Std | — | — | 0.27 (0.01, 10.79) | Moderate | 0.27 (0.01, 10.79) | Moderate |
| TXLC + Std vs Std | 0.27 (0.10, 0.64) | Moderate | — | — | 0.27 (0.10, 0.64) | Moderate |
| XZK + Std vs FDDP + Std | — | — | 0.93 (0.01, 63.26) | Low | 0.93 (0.01, 63.26) | Low |
| XZK + Std vs SBP + Std | — | — | 0.93 (0.01, 63.16) | Very low | 0.93 (0.01, 63.16) | Very low |
| XZK + Std vs GXSTC + Std | — | — | 0.84 (0.02, 22.01) | Low | 0.84 (0.02, 22.01) | Low |
| XZK + Std vs XSC + Std | — | — | 0.36 (0.00, 31.16) | Low | 0.36 (0.00, 31.16) | Low |
| XZK + Std vs Std | 0.39 (0.01, 4.85) | Moderate | — | — | 0.39 (0.01, 4.85) | Moderate |
| FDDP + Std vs SBP + Std | — | — | 1.00 (0.01, 65.47) | Low | 1.00 (0.01, 65.47) | Low |
| FDDP + Std vs GXSTC + Std | — | — | 0.90 (0.02, 23.02) | Low | 0.90 (0.02, 23.02) | Low |
| FDDP + Std vs XSC + Std | — | — | 0.39 (0.00, 32.24) | Low | 0.39 (0.00, 32.24) | Low |
| FDDP + Std vs Std | 0.42 (0.01, 5.06) | Moderate | — |  | 0.42 (0.01, 5.06) | Moderate |
| SBP + Std vs GXSTC + Std | — | — | 0.90 (0.02, 23.39) | Moderate | 0.90 (0.02, 23.39) | Moderate |
| SBP + Std vs XSC + Std | — | — | 0.39 (0.00, 32.64) | Low | 0.39 (0.00, 32.64) | Low |
| SBP + Std vs Std | 0.42 (0.01, 5.18) | Moderate | — | — | 0.42 (0.01, 5.18) | Moderate |
| GXSTC + Std vs XSC + Std |  |  | 0.44 (0.01, 23.39) | Low | 0.44 (0.01, 23.39) | Low |
| GXSTC + Std vs Std | 0.46 (0.06, 2.42) | Moderate | — | — | 0.46 (0.06, 2.42) | Moderate |
| XSC + Std vs Std | 1.00 (0.03, 38.06) | Moderate | — | — | 1.00 (0.03, 38.06) | Moderate |

## Table 7: GRADE assessments of TLR

| Comparison | Direct Estimate | Certainty | Indirect Estimate | Certainty | Network Estimate | Certainty |
| --- | --- | --- | --- | --- | --- | --- |
| CXQ + Std vs FDDP + Std | — | — | 0.91 (0.02, 37.88) | Low | 0.91 (0.02, 37.88) | Low |
| CXQ + Std vs GXSTC + Std | — | — | 0.36 (0.01, 16.66) | Low | 0.36 (0.01, 16.66) | Low |
| CXQ + Std vs TXLC + Std | — | — | 0.22 (0.01, 1.60) | Very low | 0.22 (0.01, 1.60) | Very low |
| CXQ + Std vs SBP + Std | — | — | 0.20 (0.01, 1.40) | Low | 0.20 (0.01, 1.40) | Low |
| CXQ + Std vs XSC + Std | — | — | 0.20 (0.01, 1.99) | Low | 0.20 (0.01, 1.99) | Low |
| CXQ + Std vs QYDP + Std | — | — | 0.19 (0.01, 1.57) | Moderate | 0.19 (0.01, 1.57) | Moderate |
| CXQ + Std vs DHI + Std | — | — | 0.08 (0.00, 0.78) | Low | 0.08 (0.00, 0.78) | Low |
| CXQ + Std vs XZK + Std | — | — | 0.25 (0.05, 1.08) | Low | 0.25 (0.05, 1.08) | Low |
| CXQ + Std vs Std | 0.10 (0.00, 0.60) | Moderate | — | — | 0.10 (0.00, 0.60) | Moderate |
| FDDP + Std vs GXSTC + Std | — | — | 0.39 (0.01, 18.49) | Low | 0.39 (0.01, 18.49) | Low |
| FDDP + Std vs TXLC + Std | — | — | 0.24 (0.01, 1.76) | Moderate | 0.24 (0.01, 1.76) | Moderate |
| FDDP + Std vs SBP + Std | — | — | 0.24 (0.00, 12.48) | Low | 0.24 (0.00, 12.48) | Low |
| FDDP + Std vs XSC + Std | — | — | 0.22 (0.01, 1.52) | Low | 0.22 (0.01, 1.52) | Low |
| FDDP + Std vs QYDP + Std | — | — | 0.22 (0.01, 2.16) | Low | 0.22 (0.01, 2.16) | Low |
| FDDP + Std vs DHI + Std | — | — | 0.21 (0.01, 1.73) | Low | 0.21 (0.01, 1.73) | Low |
| FDDP + Std vs XZK + Std | — | — | 0.09 (0.00, 0.84) | Low | 0.09 (0.00, 0.84) | Low |
| FDDP + Std vs Std | 0.11 (0.00, 0.65) | Moderate | — | — | 0.11 (0.00, 0.65) | Moderate |
| GXSTC + Std vs TXLC + Std | — | — | 0.59 (0.02, 6.03) | Low | 0.59 (0.02, 6.03) | Low |
| GXSTC + Std vs SBP + Std | — | — | 0.61 (0.01, 36.53) | Low | 0.61 (0.01, 36.53) | Low |
| GXSTC + Std vs XSC + Std | — | — | 0.54 (0.02, 5.31) | Low | 0.54 (0.02, 5.31) | Low |
| GXSTC + Std vs QYDP + Std | — | — | 0.54 (0.02, 7.20) | Low | 0.54 (0.02, 7.20) | Low |
| GXSTC + Std vs DHI + Std | — | — | 0.53 (0.02, 5.84) | Low | 0.53 (0.02, 5.84) | Low |
| GXSTC + Std vs XZK + Std | — | — | 0.22 (0.01, 2.81) | Very low | 0.22 (0.01, 2.81) | Very low |
| GXSTC + Std vs Std | 0.26 (0.01, 2.31) | Moderate | — | — | 0.26 (0.01, 2.31) | Moderate |
| TXLC + Std vs SBP + Std | — | — | 1.06 (0.08, 35.98) | Low | 1.06 (0.08, 35.98) | Low |
| TXLC + Std vs XSC + Std | — | — | 0.92 (0.35, 2.31) | Moderate | 0.92 (0.35, 2.31) | Moderate |
| TXLC + Std vs QYDP + Std | — | — | 0.93 (0.23, 4.15) | Low | 0.93 (0.23, 4.15) | Low |
| TXLC + Std vs DHI + Std | — | — | 0.89 (0.28, 2.91) | Low | 0.89 (0.28, 2.91) | Low |
| TXLC + Std vs XZK + Std | — | — | 0.39 (0.09, 1.54) | Low | 0.39 (0.09, 1.54) | Low |
| TXLC + Std vs Std | 0.44 (0.20, 0.87) | Moderate | — | — | 0.44 (0.20, 0.87) | Moderate |
| SBP + Std vs XSC + Std | — | — | 0.87 (0.03, 11.57) | Low | 0.87 (0.03, 11.57) | Low |
| SBP + Std vs QYDP + Std | — | — | 0.88 (0.02, 15.06) | Low | 0.88 (0.02, 15.06) | Low |
| SBP + Std vs DHI + Std | — | — | 0.84 (0.02, 12.47) | Low | 0.84 (0.02, 12.47) | Low |
| SBP + Std vs XZK + Std | — | — | 0.36 (0.01, 5.96) | Low | 0.36 (0.01, 5.96) | Low |
| SBP + Std vs Std | 0.41 (0.01, 5.11) | Moderate | — | — | 0.41 (0.01, 5.11) | Moderate |
| XSC + Std vs QYDP + Std | — | — | 1.01 (0.28, 4.29) | Low | 1.01 (0.28, 4.29) | Low |
| XSC + Std vs DHI + Std | — | — | 0.97 (0.34, 2.97) | Low | 0.97 (0.34, 2.97) | Low |
| XSC + Std vs XZK + Std | — | — | 0.42 (0.11, 1.58) | Low | 0.42 (0.11, 1.58) | Low |
| XSC + Std vs Std | 0.48 (0.26, 0.84) | Moderate | — | — | 0.48 (0.26, 0.84) | Moderate |
| QYDP + Std vs DHI + Std | — | — | 0.96 (0.20, 4.29) | Low | 0.96 (0.20, 4.29) | Low |
| QYDP + Std vs XZK + Std | — | — | 0.41 (0.07, 2.18) | Low | 0.41 (0.07, 2.18) | Low |
| QYDP + Std vs Std | 0.47 (0.12, 1.49) | Moderate | — | — | 0.47 (0.12, 1.49) | Moderate |
| DHI + Std vs XZK + Std | — | — | 0.43 (0.09, 1.90) | Very low | 0.43 (0.09, 1.90) | Very low |
| DHI + Std vs Std | 0.49 (0.19, 1.17) | Moderate | — | — | 0.49 (0.19, 1.17) | Moderate |
| XZK + Std vs Std | 1.12 (0.35, 3.85) | Moderate | — | — | 1.12 (0.35, 3.85) | Moderate |

# Supplementary Figures

## Supplementary Figure 1: Trace and Density plots


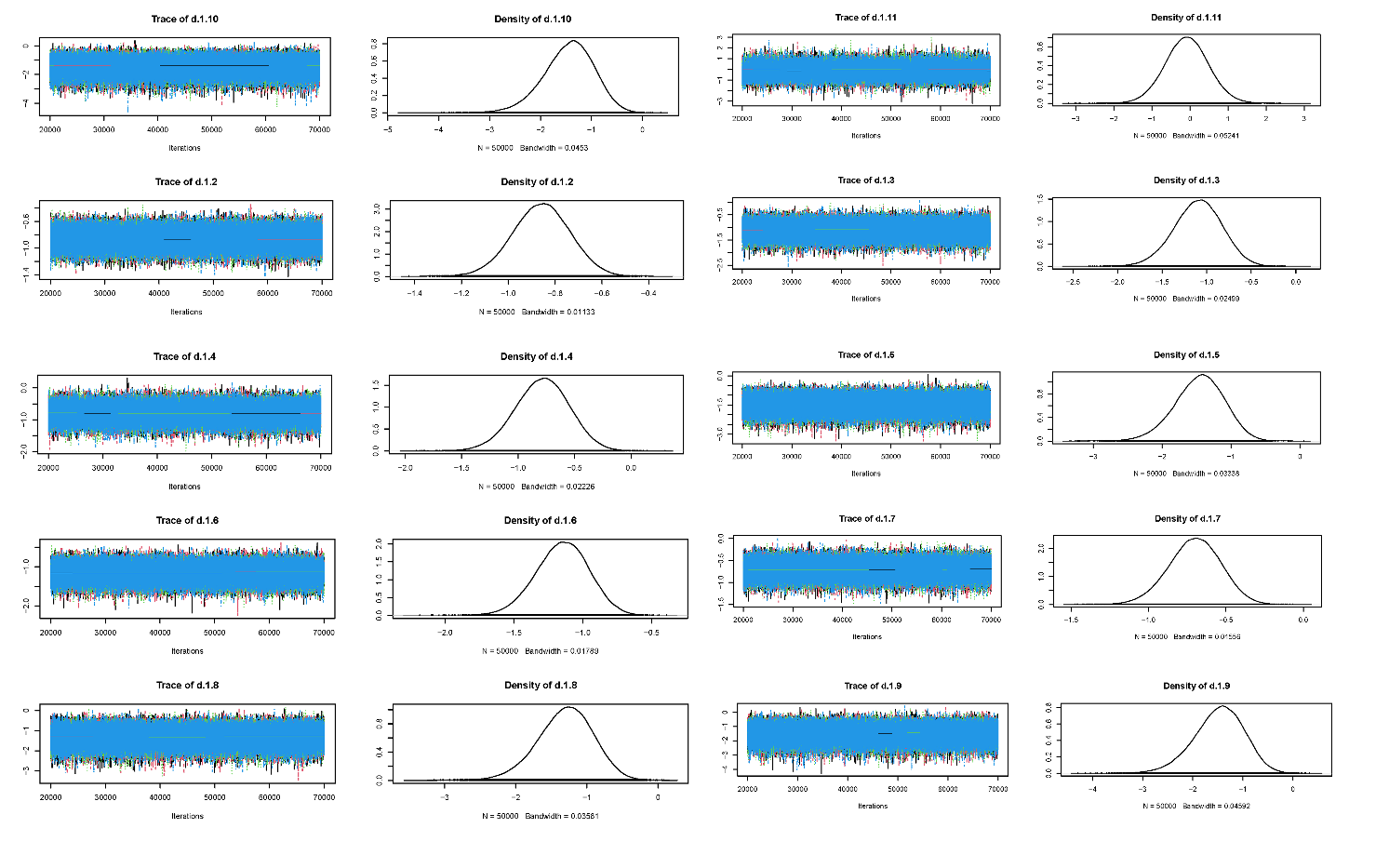


Models were calculated by generating 50,000 sample iterations with an initial burn-in period of 20,000 iterations.

## Supplementary Figure 2: Brooks-Gelman-Rubin diagnostics plot
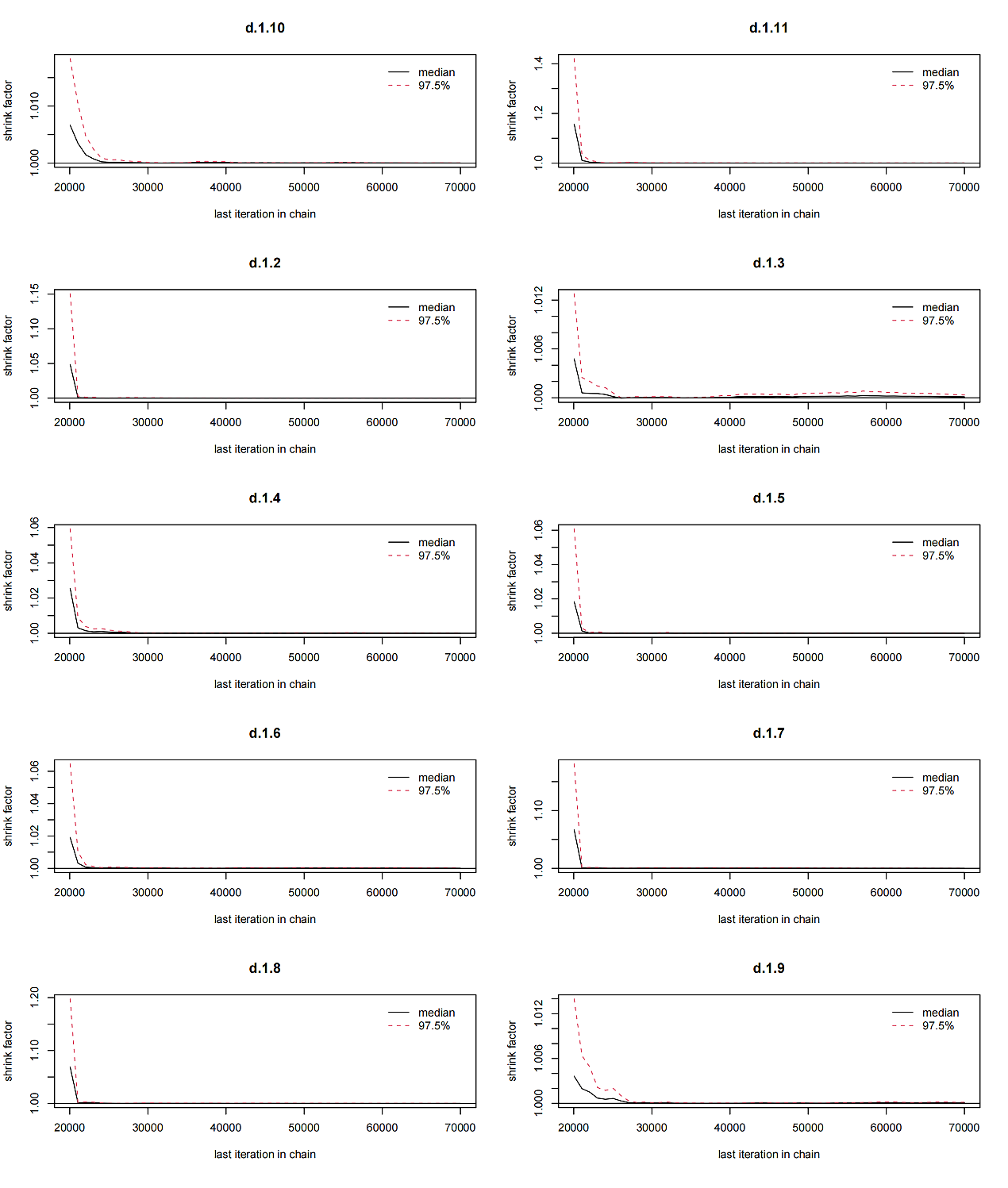


Models were calculated by generating 50,000 sample iterations with an initial burn-in period of 20,000 iterations.

## Supplementary Figure 3: Forest plot for preventing angiographic restenosis of 10 Chinese patent medicines compared to Std


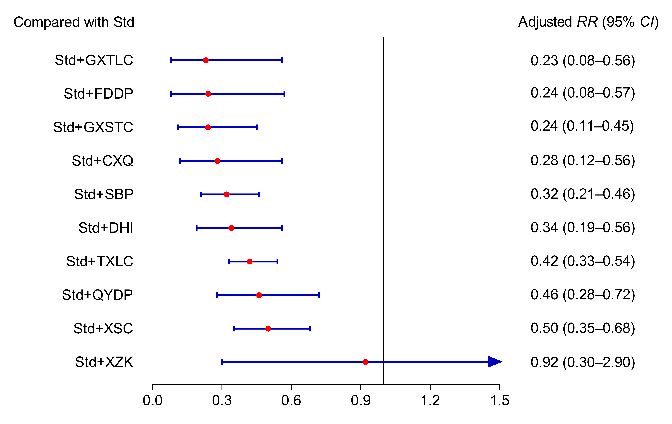


## Supplementary Figure 4: Forest plot for preventing recurrence angina of 8 Chinese patent medicine compared to Std


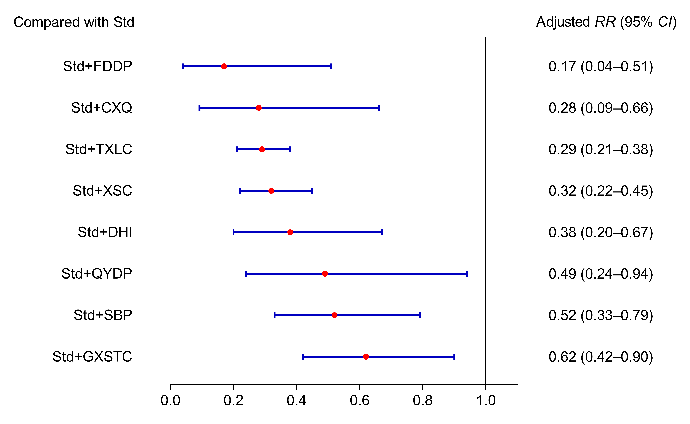


## Supplementary Figure 5: Forest plot for preventing acute myocardial infarction of 8 Chinese patent medicine compared to Std


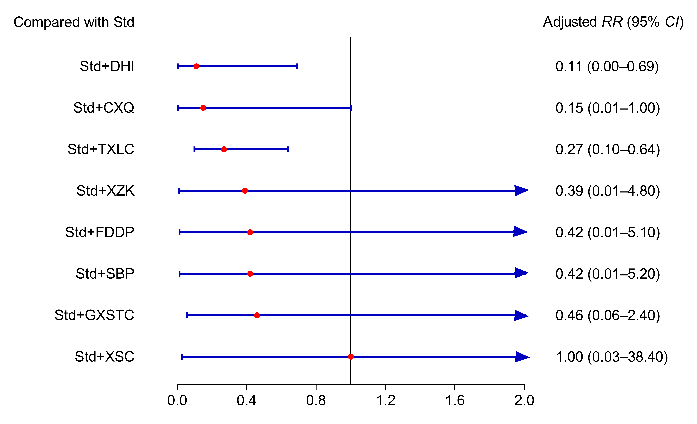


## Supplementary Figure 6: Forest plot for preventing target lesion revascularization of 9 Chinese patent medicine compared to Std


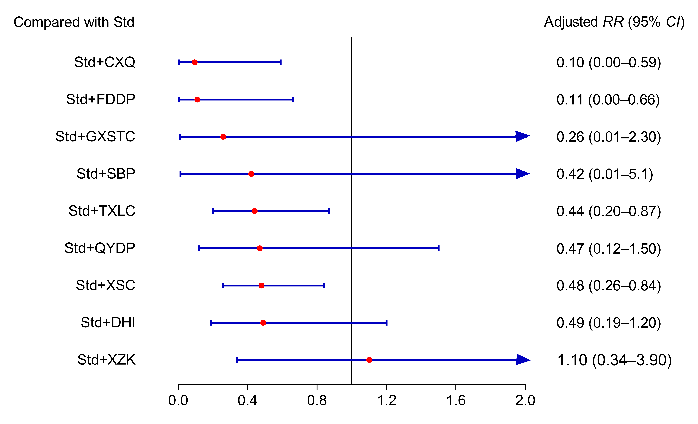


## Supplementary Figure 7: Results of subgroup analyses (studies which number of patients ≥100)


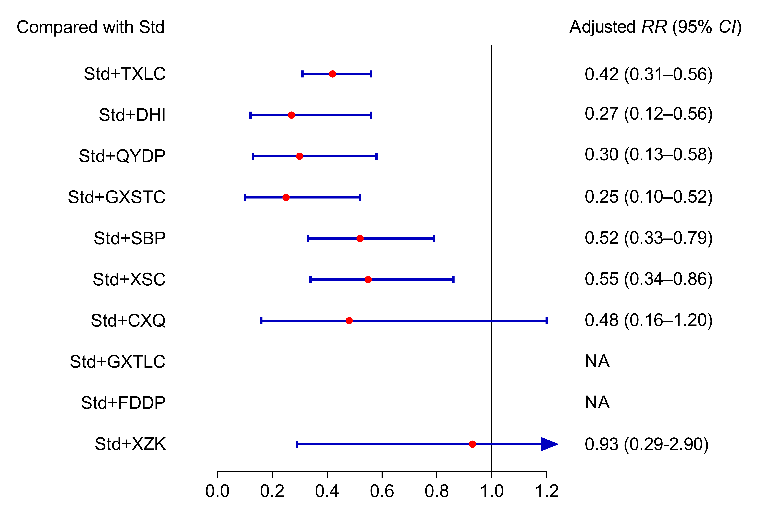


## Supplementary Figure 8: Results of subgroup analyses (studies which number of patients <100)


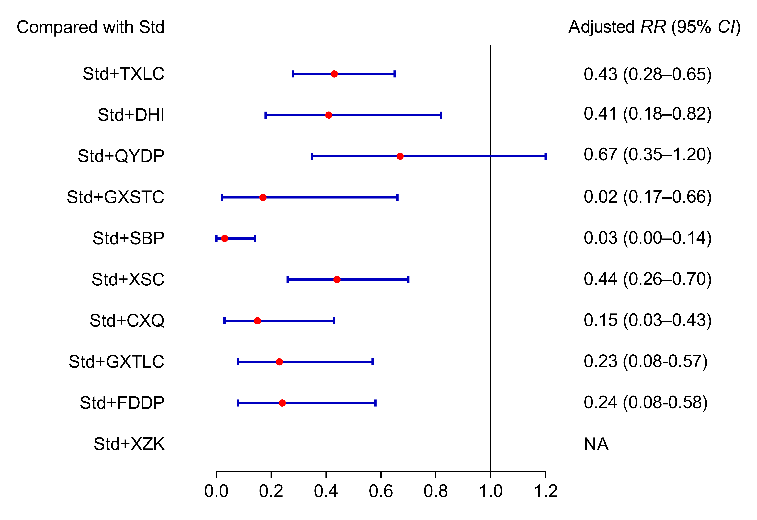


## Supplementary Figure 9: sensitivity analysis, outcome: angiographic restenosis
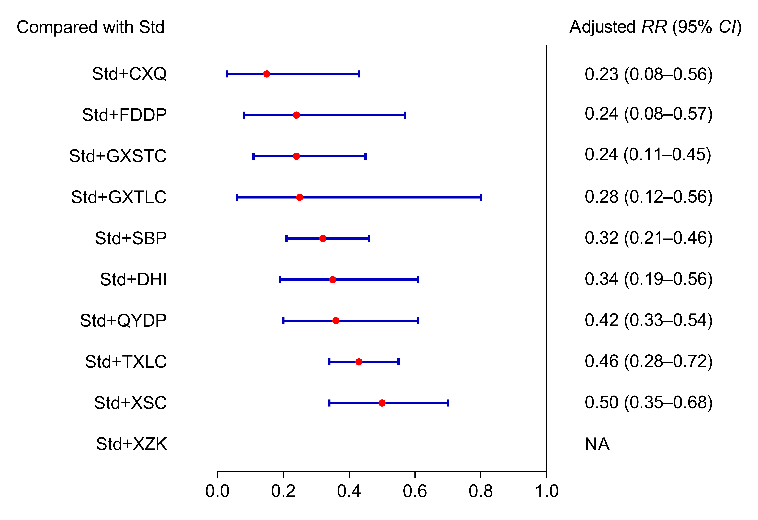


## Supplementary Figure 10: sensitivity analysis, outcome: recurrence angina


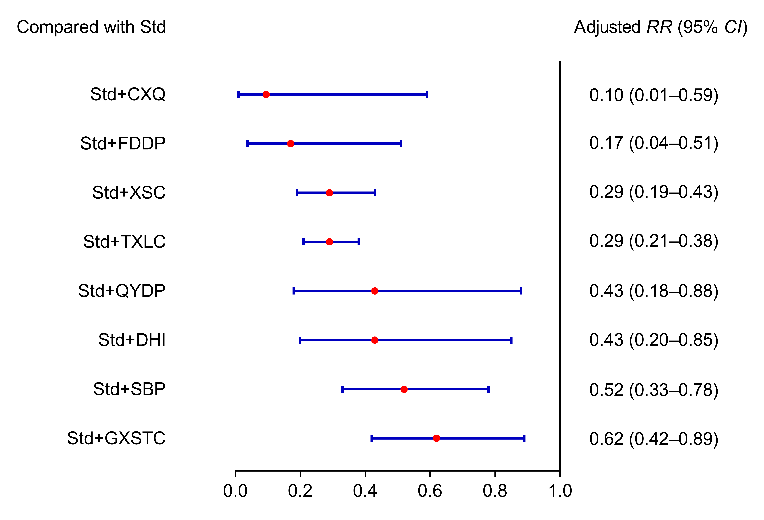


## Supplementary Figure 11: sensitivity analysis, outcome: acute myocardial infarction


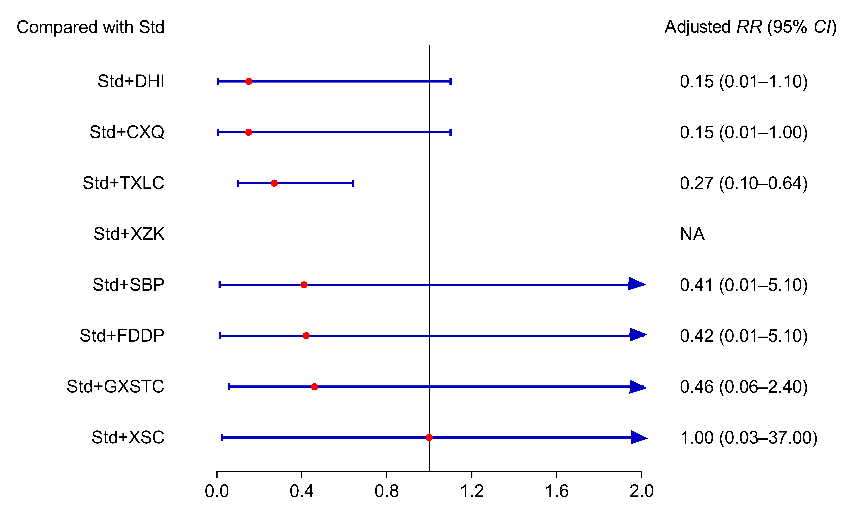


## Supplementary Figure 12: sensitivity analysis, outcome: target lesion revascularization


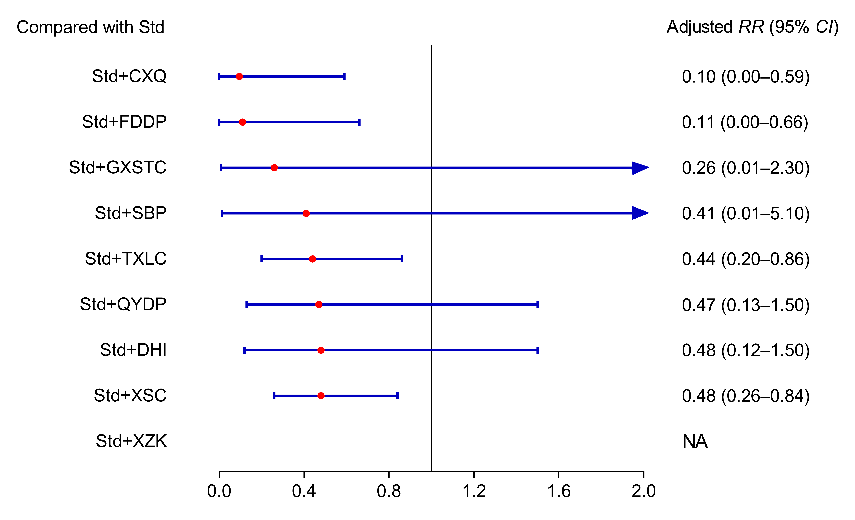


## Supplementary Figure 13: Comparison-specific funnel chart in terms of efficiency outcomes


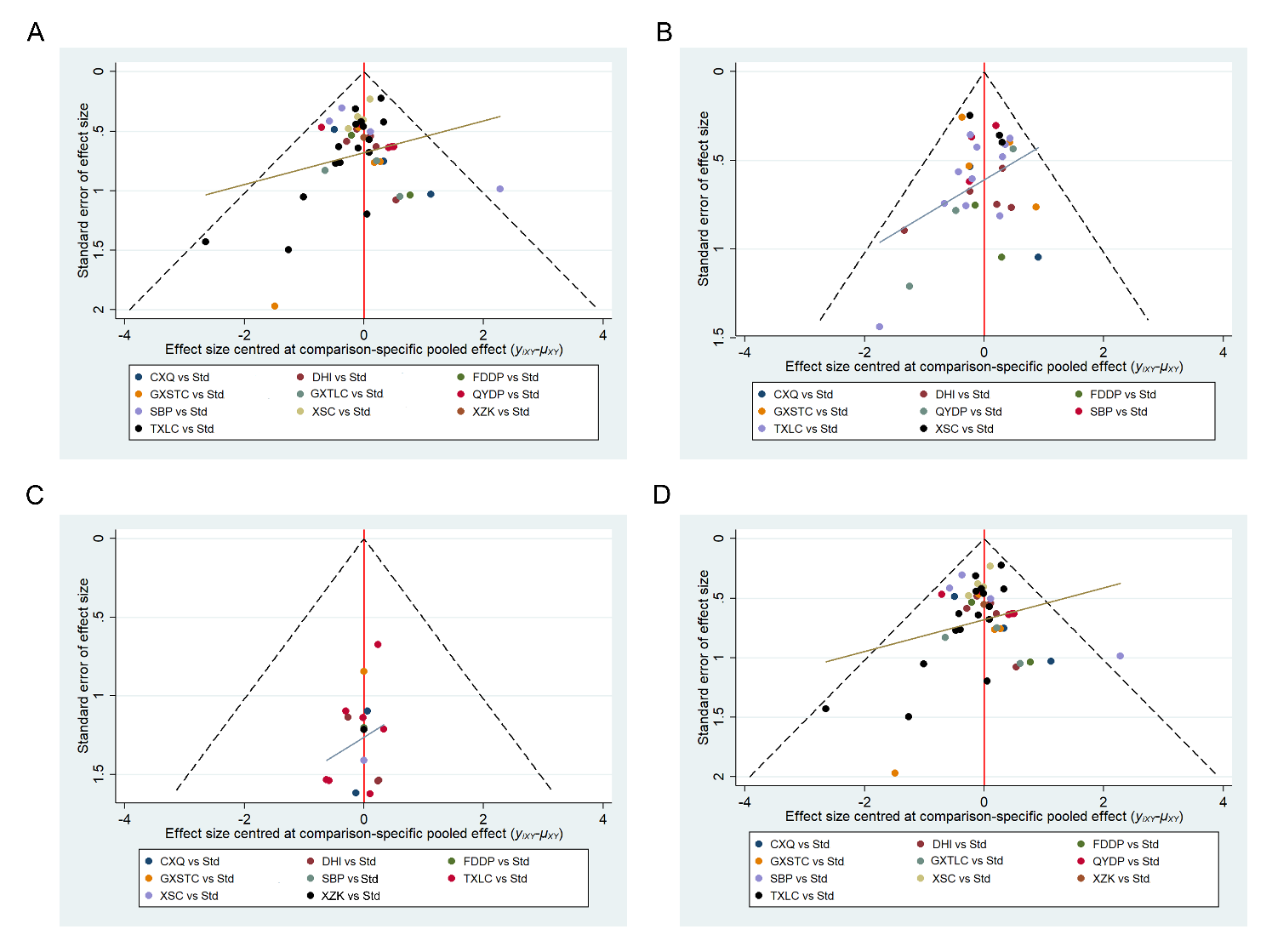


1. The outcome of angiographic restenosis; (B) The outcome of recurrence angina; (C) The outcome of acute myocardial infarction; (D) The outcome of target lesion revascularization.

CXQ: Chuanxiongqin tablet; DHI: Danhong injection; FDDP: Fufang Danshen dripping pill; GXSTC: Guanxin Shutong capsule; GXTLC: Guanxin Tongluo capsule; QYDP: Qishen Yiqi dripping pill; SBP: Shexiang Baoxin pill; Std: standard treatment; TXLC: Tongxinluo capsule; XSC: Xiongshao capsule; XZK: Xuezhikang capsule.

# Search strategies

## Text 1: Pubmed

#1: "medicine, Chinese traditional"[Mesh]

#2: "TCM" OR "traditional Chinese medicine" OR "traditional Chinese herbal medicine" OR "Chung I Hsueh" OR "Hsueh, Chung I" OR "traditional medicine, Chinese" OR "Zhong Yi Xue" OR "Chinese traditional medicine" OR "Chinese medicine, traditional" OR "traditional Tongue diagnosis" OR "Tongue diagnoses, traditional" OR "Tongue diagnosis, traditional" OR "traditional Tongue diagnoses" OR "traditional Tongue assessment" OR "Tongue assessment, traditional" OR "traditional Tongue assessments" OR "drugs, Chinese herbal" OR "plant extracts, Chinese" OR "Chinese plant extracts" OR "extracts, Chinese plant" OR "Chinese herbal drugs" OR "Chinese drugs, plant" OR "herbal drugs, Chinese" OR "medicinal plant" OR "plant, medicinal" OR "medicinal plants" OR "medicinal herbs" OR "herb, medicinal" OR "medicinal herb" OR "herbs, medicinal" OR "herbal medicine" OR "herbal" OR "herb" OR "pharmaceutical plants" OR "pharmaceutical plant" OR "plant, pharmaceutical" OR "plants, pharmaceutical" OR "healing plants" OR "healing plant" OR "plant, healing" OR "plants, healing" OR "complementary" OR "alternative medicine"

#3: #1 OR #2

#4: "percutaneous coronary intervention"[Mesh]

#5: "coronary intervention, percutaneous" OR "coronary interventions, percutaneous" OR "intervention, percutaneous coronary" OR "interventions, percutaneous coronary" OR "percutaneous coronary interventions" OR "percutaneous coronary revascularization" OR "coronary revascularization, percutaneous" OR "coronary revascularizations, percutaneous" OR "percutaneous coronary revascularizations" OR "revascularization, percutaneous coronary" OR "revascularizations, percutaneous coronary" OR "stent" OR "balloon" OR "PCI" OR "PTCA" OR "percutaneous transluminal coronary angioplasty"

#6: #4 OR #5

#7: "restenoses" OR "restenosis" OR "stricture"

#8: #3 AND #6 AND #7

## Text 2: Cochrane Library

#1: "medicine, Chinese traditional"[Mesh]

#2: "TCM" OR "traditional Chinese medicine" OR "traditional Chinese herbal medicine" OR "Chung I Hsueh" OR "Hsueh, Chung I" OR "traditional medicine, Chinese" OR "Zhong Yi Xue" OR "Chinese traditional medicine" OR "Chinese medicine, traditional" OR "traditional Tongue diagnosis" OR "Tongue diagnoses, traditional" OR "Tongue diagnosis, traditional" OR "traditional Tongue diagnoses" OR "traditional Tongue assessment" OR "Tongue assessment, traditional" OR "traditional Tongue assessments" OR "drugs, Chinese herbal" OR "plant extracts, Chinese" OR "Chinese plant extracts" OR "extracts, Chinese plant" OR "Chinese herbal drugs" OR "Chinese drugs, plant" OR "herbal drugs, Chinese" OR "medicinal plant" OR "plant, medicinal" OR "medicinal plants" OR "medicinal herbs" OR "herb, medicinal" OR "medicinal herb" OR "herbs, medicinal" OR "herbal medicine" OR "herbal" OR "herb" OR "pharmaceutical plants" OR "pharmaceutical plant" OR "plant, pharmaceutical" OR "plants, pharmaceutical" OR "healing plants" OR "healing plant" OR "plant, healing" OR "plants, healing" OR "complementary" OR "alternative medicine"

#3: #1 OR #2

#4: "percutaneous coronary intervention"[Mesh]

#5: "coronary intervention, percutaneous" OR "coronary interventions, percutaneous" OR "intervention, percutaneous coronary" OR "interventions, percutaneous coronary" OR "percutaneous coronary interventions" OR "percutaneous coronary revascularization" OR "coronary revascularization, percutaneous" OR "coronary revascularizations, percutaneous" OR "percutaneous coronary revascularizations" OR "revascularization, percutaneous coronary" OR "revascularizations, percutaneous coronary" OR "stent" OR "balloon" OR "PCI" OR "PTCA" OR "percutaneous transluminal coronary angioplasty"

#6: #4 OR #5

#7: "restenoses" OR "restenosis" OR "stricture"

#8: #3 AND #6 AND #7

## Text 3: Web of Science

#1: "medicine, Chinese traditional" OR "TCM" OR "traditional Chinese medicine" OR "traditional Chinese herbal medicine" OR "Chung I Hsueh" OR "Hsueh, Chung I" OR "traditional medicine, Chinese" OR "Zhong Yi Xue" OR "Chinese traditional medicine" OR "Chinese medicine, traditional" OR "traditional Tongue diagnosis" OR "Tongue diagnoses, traditional" OR "Tongue diagnosis, traditional" OR "traditional Tongue diagnoses" OR "traditional Tongue assessment" OR "Tongue assessment, traditional" OR "traditional Tongue assessments" OR "drugs, Chinese herbal" OR "plant extracts, Chinese" OR "Chinese plant extracts" OR "extracts, Chinese plant" OR "Chinese herbal drugs" OR "Chinese drugs, plant" OR "herbal drugs, Chinese" OR "medicinal plant" OR "plant, medicinal" OR "medicinal plants" OR "medicinal herbs" OR "herb, medicinal" OR "medicinal herb" OR "herbs, medicinal" OR "herbal medicine" OR "herbal" OR "herb" OR "pharmaceutical plants" OR "pharmaceutical plant" OR "plant, pharmaceutical" OR "plants, pharmaceutical" OR "healing plants" OR "healing plant" OR "plant, healing" OR "plants, healing" OR "complementary" OR "alternative medicine"

#2: "percutaneous coronary intervention" OR "coronary intervention, percutaneous" OR "coronary interventions, percutaneous" OR "intervention, percutaneous coronary" OR "interventions, percutaneous coronary" OR "percutaneous coronary interventions" OR "percutaneous coronary revascularization" OR "coronary revascularization, percutaneous" OR "coronary revascularizations, percutaneous" OR "percutaneous coronary revascularizations" OR "revascularization, percutaneous coronary" OR "revascularizations, percutaneous coronary" OR "stent" OR "balloon" OR "PCI" OR "PTCA" OR "percutaneous transluminal coronary angioplasty"

#3: "restenoses" OR "restenosis" OR "stricture"

#4: #1 AND #2 AND #3

## Text 4: Embase

("medicine, Chinese traditional" OR "TCM" OR "traditional Chinese medicine" OR "traditional Chinese herbal medicine" OR "Chung I Hsueh" OR "Hsueh, Chung I" OR "traditional medicine, Chinese" OR "Zhong Yi Xue" OR "Chinese traditional medicine" OR "Chinese medicine, traditional" OR "traditional Tongue diagnosis" OR "Tongue diagnoses, traditional" OR "Tongue diagnosis, traditional" OR "traditional Tongue diagnoses" OR "traditional Tongue assessment" OR "Tongue assessment, traditional" OR "traditional Tongue assessments" OR "drugs, Chinese herbal" OR "plant extracts, Chinese" OR "Chinese plant extracts" OR "extracts, Chinese plant" OR "Chinese herbal drugs" OR "Chinese drugs, plant" OR "herbal drugs, Chinese" OR "medicinal plant" OR "plant, medicinal" OR "medicinal plants" OR "medicinal herbs" OR "herb, medicinal" OR "medicinal herb" OR "herbs, medicinal" OR "herbal medicine" OR "herbal" OR "herb" OR "pharmaceutical plants" OR "pharmaceutical plant" OR "plant, pharmaceutical" OR "plants, pharmaceutical" OR "healing plants" OR "healing plant" OR "plant, healing" OR "plants, healing" OR "complementary" OR "alternative medicine") AND ("percutaneous coronary intervention" OR "coronary intervention, percutaneous" OR "coronary interventions, percutaneous" OR "intervention, percutaneous coronary" OR "interventions, percutaneous coronary" OR "percutaneous coronary interventions" OR "percutaneous coronary revascularization" OR "coronary revascularization, percutaneous" OR "coronary revascularizations, percutaneous" OR "percutaneous coronary revascularizations" OR "revascularization, percutaneous coronary" OR "revascularizations, percutaneous coronary" OR "stent" OR "balloon" OR "PCI" OR "PTCA" OR "percutaneous transluminal coronary angioplasty") AND ("restenoses" OR "restenosis" OR "stricture")
